# Bi-objective goal programming for balancing costs vs. nutritional adequacy

**DOI:** 10.3389/fnut.2022.1056205

**Published:** 2022-12-15

**Authors:** Melissa F. Koenen, Marleen Balvert, Hein Fleuren

**Affiliations:** ^1^Zero Hunger Lab, Tilburg School of Economics and Management, Tilburg University, Tilburg, Netherlands; ^2^Department of Econometrics and Operations Research, Tilburg School of Economics and Management, Tilburg University, Tilburg, Netherlands

**Keywords:** diet optimization, goal programming, bi-objective, nutritional adequacy, linear programming

## Abstract

**Introduction:**

Linear programming (LP) is often used within diet optimization to find, from a set of available food commodities, the most affordable diet that meets the nutritional requirements of an individual or (sub)population. It is, however, not always possible to create a feasible diet, as certain nutritional requirements are difficult to meet. In that case, goal programming (GP) can be used to minimize deviations from the nutritional requirements in order to obtain a near feasible diet. With GP the cost of the diet is often overlooked or taken into account using the ε-constraint method. This method does not guarantee to find all possible trade-offs between costs and nutritional deficiency without solving many uninformative LPs.

**Methods:**

We present a method to find all trade-offs between any two linear objectives in a dietary LP context that is simple, does not solve uninformative LPs and does not need prior input from the decision maker (DM). This method is a bi-objective algorithm based on the NonInferior Set Estimation (NISE) method that finds all efficient trade-offs between two linear objectives.

**Results:**

In order to show what type of insights can be gained from this approach, two analyses are presented that investigate the relation between cost and nutritional adequacy. In the first analysis a diet with a restriction on the exact energy intake is considered where all nutrient intakes except energy are allowed to deviate from their prescription. This analysis is especially helpful in case of a restrictive budget or when a nutritionally adequate diet is either unaffordable or unattainable. The second analysis only relaxes the exact energy intake, where the other nutrients are kept within their requirements, to investigate how the energy intake affects the cost of a diet. Here, we describe in what situations the so-called more-for-less paradox takes place, which can be induced by requiring an exact energy intake.

**Conclusion:**

To the best of our knowledge, we are the first to address how to obtain all efficient trade-offs of two linear objectives in a dietary LP context and how this can be used for analyses.

## 1. Introduction

The importance of a nutritionally adequate diet has been widely studied and acknowledged (1, 2). The adverse effects of malnutrition can result in acute and/or lifelong problems affecting the health and well-being of an individual. A micronutrient deficiency indicates a lack of essential vitamins and minerals, and is highly prevalent among young children and pregnant females in low- and middle-income countries (3). It is estimated that in 2004 the deficiencies of vitamin A and zinc were responsible for the deaths of 0.6 million and 0.4 million children, respectively, and the deficiency of iron was responsible for 0.1 million maternal deaths (4). These detrimental effects of nutritional deficiencies are caused by a combination of insufficient availability of affordable and nutritious foods (5) and a relatively high micronutrient demand of young children, adolescent girls and pregnant females.

Optimization techniques such as linear programming (LP) can help to find a nutritionally adequate diet. In a dietary context LP is often applied to find the most affordable diet for an individual, subpopulation or population given a set of available food commodities and nutritional requirements (6–9). We focus on dietary optimization in a humanitarian context, where diet optimization is used to determine the absolute minimum cost of a nutritious diet. This can then be compared to a household income to assess whether the inhabitants of a certain region can afford a healthy diet and which (governmental) interventions are necessary (5). While menu planning is beyond the scope of this paper, the discussed methods and analyses can readily be extended.

The key factor of the described LP is that the resulting diet is the cheapest diet that is nutritionally adequate. However, it is not always possible to construct a nutritionally adequate diet. For example, in low- and middle-income countries it is challenging to meet the nutritional requirements with the foods available, as there often is a shortage of (affordable) nutrient-dense foods. This is even more problematic when diseases are prevalent that impact the intake and absorption of nutrients (10). An additional challenge is the inclusion of dietary and cultural preferences, which further limits the diet composition. It is for example difficult to compose a vegan nutrient adequate diet, as the primary source of vitamin B12 is found nearly only in animal source foods such as meat and dairy (1).

When it is not possible to obtain a feasible diet, there is a need to find a diet that is “somewhat close” to feasible while still taking costs into consideration. As an example, a nutritious diet may be unaffordable due to an insufficient household budget. This is a real-life constraint: before COVID-19 already 70% of the people in low- and middle-income countries could not afford a healthy diet (11). A most recent report (12) estimates that in 2020 almost 3.1 billion people worldwide could not afford a healthy meal. In this case it is still necessary to find a diet that is as nutritious as possible within the budgetary restrictions. This is particularly useful for individuals whose nutritional requirements are challenging. For example, for adolescent girls it is difficult to obtain a nutritious and affordable diet as they require more iron while their energy intake is lower than for boys of the same age (13). Thus, adolescent girls need more (relatively expensive) nutrient-dense foods which might be unavailable.

The question is how to define what “somewhat close” and “second best” entails. Goal programming (GP) is an often used approach (14–16) for finding solutions that are somewhat close to feasible, where additional decision variables are introduced to measure the deviation from minimum and maximum requirements for each nutritional constraint. The (weighted) summed deviation of all (relevant) nutrients is minimized. In case a nutritionally adequate diet is possible, the summed deviation will be zero. Otherwise, it will present a “second best” diet with minimal summed deviation.

Although most GP approaches entirely omit the original cost objective (14, 16), the costs are included in the model in (15) through both a cost constraint and extending the GP objective to minimize a deviation of the cost from the optimal cost. The trade-off between the cost and the nutritional adequacy—which hinges on the fact that a healthier diet costs more, discussed in much detail in the overview paper of (17)—is reflected by weights assigned to the nutritional deviations and the cost. The disadvantage of this approach is that the decision maker (DM) has to specify weights for each of the objectives indicating their preference. These weights are generally difficult to interpret and even more difficult to set a priori. Instead, it is more beneficial to supply the DM with several solutions that each represent a different trade-off between cost and nutritional adequacy such that they can select the solution that suites them best. Furthermore, the complete overview of possible trade-offs allows the DM to analyze and understand how these objectives affect each other. The most accurate description of the relation between the objectives is obtained when all possible trade-offs are found.

In order to obtain all trade-offs one could progressively tighten the cost constraint (18). This method is known as the ε-constraint method (19), where ε denotes the increment that tightens the constraint. Various papers in the current literature use the ε-constraint method to find trade-offs between two linear objectives in a dietary context (18, 20–24). With the ε-constraint method one has to make a selection for ε. Unless the increments for ε are sufficiently small, one cannot obtain all trade-offs. To determine how small the increments of ε should be to obtain all trade-offs, one needs to know all trade-offs, which brings us back to the issue of how to find all trade-offs. Of course, simply decreasing the size of ε usually results in a more accurate representation of the trade-offs, but it also implies that many uninformative LPs that do not result in a new trade-off point have to be solved.

An alternative method is weighted sum scalarization (25), where each of the objectives gets assigned a certain weight within the combined objective. Selecting different weights potentially yields different trade-offs. Similar to the problem of the ε-constraint method, one needs to select these weights. There are ways to make reasonable selections for these weights, e.g., (26), but they do not necessarily result in all trade-offs. Selecting only sufficiently small changes in the weights again results in a more accurate representation of the trade-offs, but it also implies that many uninformative LPs are solved.

Therefore, in this paper we focus on finding all trade-offs between two linear objectives in a dietary LP context, where the method should be simple, does not solve uninformative LPs and does not need prior input from the DM. To the best of our knowledge, we are the first to address how to obtain all efficient trade-offs of two linear objectives in a dietary LP context. Our contributions are threefold. First, we illustrate multi-objective optimization concepts such as *efficiency* and *Pareto curve* in a dietary context. We point out the importance of finding all efficient trade-offs, as they help to make decisions when there is a restrictive budget, or when a nutritionally adequate diet is either unaffordable or unattainable. Second, we present a bi-objective algorithm which is able to obtain all efficient trade-offs of two linear objectives. This method is based on the principles of the NISE (NonInferior Set Estimation) method (27). Here, we do not need an a priori preference of the DM for how to trade off the objectives. Third, we explain what types of insights can be gained using the bi-objective approach by performing two analyses that are relevant when studying the nutritional adequacy and cost of a diet. In the first analysis an exact amount for energy intake is considered, where all nutrients except energy are allowed to deviate. The analysis shows whether a nutritious diet is attainable and what the nutritional content of a diet is given a particular budget. In the second analysis we investigate how the energy intake impacts the cost of a diet by relaxing the energy intake and keeping all other nutrients within their requirements.

Our paper is structured in the following way: in Section 2 we formally introduce the linear diet optimization problem (LDOP). In Section 3.1 we explain how to apply GP to measure nutritional inadequacies. In Section 3.2 we describe multi-objective concepts in a dietary context and we show the bi-objective algorithm that obtains all efficient trade-offs. In Section 4 we explain what types of insights can be obtained from this procedure by presenting the two described analyses. Finally, Section 5 provides a discussion.

## 2. Preliminaries

In this section we describe the LDOP, which is modeled using LP. We use this formulation as a starting point for the remainder of this work. The reader is referred to (28) for more information regarding general LP optimization.

The LDOP is a traditional LP problem (29, 30), which aims to find the most affordable diet for an individual given a set of available food commodities and the individual's nutritional requirements. The decision variable *x*_*i*_ states the amount of food commodity *i* included in the diet, given a set of available food commodities I. The total cost of a diet is determined by summing over the cost of each included food commodity, where the cost *c*_*i*_ per unit of food commodity *i* is known. As the diet should adhere to nutritional requirements, lower and upper limits are provided for each nutrient *n* ∈ N indicated by ${\underline{b}}_{n}$ and ${\bar{b}}_{n}$, respectively. Note that in practice not all nutrients have an upper bound. The amount of nutrient *n* per unit of food commodity *i* is represented by $a_{i}^{n}$. The LDOP is then formally described as


(2.1) $$\begin{array}{l} (\text{LDOP})=\min\sum_{i\in ℐ}c_{i}x_{i} \end{array}$$

(2.2) $$\begin{array}{l} \text{ s.t.}\sum_{i\in ℐ}a_{i}^{n}x_{i}\geq {\underline{b}}_{n}\;\forall n\in N \end{array}$$

(2.3) $$\begin{array}{l} \;\sum_{i\in ℐ}a_{i}^{n}x_{i}\leq {\bar{b}}_{n}\;\forall n\in N \end{array}$$

(2.4) $$\begin{array}{l} \;x_{i}\geq 0\;\forall i\in ℐ, \end{array}$$

where the Equation (2.1) minimizes the cost of the diet, Equations (2.2), (2.3) ensure the nutritional feasibility and constraints Equation (2.4) makes sure that only non-negative amounts are included in the diet.

In the LDOP the energy intake of an individual is considered a nutrient which has to adhere to certain requirements. Originally, the energy intake was modeled as a minimum requirement only (31–33). However, this can lead to unacceptable diets consisting of too many calories, as energy-dense foods are relatively cheap compared to nutrient-dense foods. Additionally, this may lead to diets which consist of too large amounts of food to consume. This can be overcome by either including a range for the energy constraint (34–36), or by setting the energy intake to an exact amount (6–9, 16, 18, 37, 38). In line with recent literature we will initially consider an exact energy intake. In Section 4.2 however we relax this assumption to investigate how the energy intake influences the cost of a diet.

Besides these nutritional constraints that define the core of the LDOP, often additional constraints are included to increase the diversity and palatability of a diet, e.g., food commodity constraints (28, 35), palatability constraints (35) or food group constraints (28). As this is not the main focus of this work, we only include food commodity constraints to impose a minimum and maximum intake on each food commodity. These are structured as follows


(2.5) $$\begin{array}{l} x_{i}\geq {\underline{d}}_{i}\;\forall i\in ℐ \end{array}$$

(2.6) $$\begin{array}{l} x_{i}\leq {\bar{d}}_{i}\;\forall i\in ℐ, \end{array}$$

where ${\underline{d}}_{i}$ and ${\bar{d}}_{i}$ denote the minimum and maximum intake of food commodity *i*, respectively.

## 3. Modeling trade-off costs vs. nutritional adequacy

This section is organized as follows. In Section 3.1 we show how GP can be used to model nutritional inadequacy with the LDOP as example. In case it is impossible to obtain a nutritionally adequate diet, GP can help to find a diet which is “somewhat close” to a nutritionally adequate diet. We present three so-called achievement functions (objectives) which are commonly used in the literature. As GP models often focus on the satisficing side of a problem [“*only […] minimizing the non-achievement of several goals*” (39)], we will initially ignore the cost objective in Section 3.1. Next, in Section 3.2 we extend the GP approach by reintroducing the diet's cost. This inherently leads to a bi-objective optimization problem, where one needs to trade off cost vs. nutritional adequacy. We present a procedure based on the NISE method (27), which efficiently computes all Pareto efficient trade-offs between cost and nutritional adequacy.

### 3.1. Minimizing nutritional inadequacy through goal programming

In order to allow for deviations of the nutritional Equations (2.2), (2.3), two additional types of variables ${\underline{z}}_{n}$ and ${\bar{z}}_{n}$, ∀*n* ∈ N are introduced that reflect the under- and overconsumption of a nutrient, respectively. There are two cases possible: either no relaxation of a nutritional constraint is needed — implying that both ${\underline{z}}_{n}$ and ${\bar{z}}_{n}$ are set to 0 — or relaxation is needed for either the lower or the upper limit. Summarized,


$$\begin{array}{l} \{\begin{array}{ll} {\underline{z}}_{n}={\bar{z}}_{n}=0 & \text{if no limit of }n\text{ is relaxed};\text{ nutrient adequacy},\\ {\underline{z}}_{n}>0,{\bar{z}}_{n}=0 & \begin{array}{l} \text{if the lower limit of }n\text{ is relaxed};\\ \text{nutrient deficiency}, \end{array}\\ {\underline{z}}_{n}=0,{\bar{z}}_{n}>0 & \begin{array}{l} \text{if the upper limit of }n\text{ is relaxed};\\ \text{nutrient excess}. \end{array} \end{array} \end{array}$$

Directly comparing the absolute nutrient excess or deficiency of one nutrient with another would lead to a larger emphasis on nutrients with a larger order of magnitude, therefore we use the absolute percentage difference for each nutrient relative to its limits. That is, for differences with regard to the upper limit we consider $|\frac{{\bar{z}}_{n}}{{\bar{b}}_{n}}|=\frac{{\bar{z}}_{n}}{{\bar{b}}_{n}}$, given that there is an upper limit for this specific nutrient. For differences with regard to the lower limit we consider $|\frac{-{\underline{z}}_{n}}{{\underline{b}}_{n}}|=\frac{{\underline{z}}_{n}}{{\underline{b}}_{n}}$. Note that deviations from the lower limit are penalized more heavily than deviations from the upper limit as ${\bar{b}}_{n}>{\underline{b}}_{n}$.

In GP convention the objectives are referred to as achievement functions. Here, they aim to minimize nutrient deficiency and excess. We consider the following three commonly used achievement functions:

1. Summed deviation of all nutrients (15, 16, 32, 39);
2. MinMax or worst-case deviation over all nutrients (15, 16, 32, 39);
3. Number of unmet nutritional constraints (40).

Below we describe how to model these three options where at this stage we only include constraints for the nutrients. Additional non-relaxed constraints such as food commodity constraints can be added upon preference of the user.

#### 3.1.1. Summed deviation

The summed deviation, or Archimedean GP, minimizes the sum of all deviations. The model is described as:


(3.1) $$\begin{array}{ll} \left(\text{GP-SD}\right)=\min & \underset{n\in N}{\sum }\left(\frac{{\underline{z}}_{n}}{{\underline{b}}_{n}}+\frac{{\bar{z}}_{n}}{{\bar{b}}_{n}}\right) \end{array}$$

(3.2) $$\begin{array}{lll} \text{s.t.} & \underset{i\in I}{\sum }a_{i}^{n}x_{i}\geq {\underline{b}}_{n}-{\underline{z}}_{n} & \forall n\in N \end{array}$$

(3.3) $$\begin{array}{ll} \underset{i\in I}{\sum }a_{i}^{n}x_{i}\leq {\bar{b}}_{n}+{\bar{z}}_{n} & \forall n\in N \end{array}$$

(3.4) $$\begin{array}{ll} {\underline{z}}_{n},{\bar{z}}_{n}\geq 0 & \forall n\in N \end{array}$$

(3.5) $$\begin{array}{ll} x_{i}\geq 0 & \forall i\in I, \end{array}$$

where the objective contains the minimization of both ${\underline{z}}_{n}$ and ${\bar{z}}_{n}$ as both under- and overconsumption are undesirable. For nutrients where only underconsumption (overconsumption) is considered, e.g., by recommendations of EFSA, FAO/WHO or IOM, only ${\underline{z}}_{n}$ (${\bar{z}}_{n}$) has to be included in the objective. Note that one can include weights in the objective to indicate the relative importance of a nutrient.

#### 3.1.2. MinMax

The MinMax or Chebyshev achievement function minimizes the deviation of the worst performing nutrient and is modeled as follows:


$$\begin{array}{l} (\text{GP-MinMax})=\min z^{\text{MM}}\\ \text{ s.t. }\left(3.2\right)-\left(3.5\right)\\ \;z^{\text{MM}}\geq \frac{{\underline{z}}_{n}}{{\underline{b}}_{n}}\;\forall n\in N\\ \;z^{\text{MM}}\geq \frac{{\bar{z}}_{n}}{{\bar{b}}_{n}}\;\forall n\in N, \end{array}$$

where an additional decision variable *z*^MM^ is introduced which denotes the percentage difference of the worst performing nutrient. Note that *z*^MM^ ≥ 0, as ${\underline{z}}_{n},{\bar{z}}_{n},{\underline{b}}_{n},{\bar{b}}_{n}\geq 0,\forall n\in N$.

#### 3.1.3. Number of unmet constraints

The third achievement function is to minimize the number of unmet constraints. For this a binary decision variable needs to be introduced for each nutrient which makes it a mixed-integer linear programming model. Let ${\underline{p}}_{n}$ be 1 in case there is a deficiency of nutrient *n* and 0 otherwise. A similar binary variable is introduced for a nutrient excess, ${\bar{p}}_{n}$. The corresponding model is:


$$\begin{array}{l} (\text{GP-Unmet})=\min\sum_{n\in N}\left({\underline{p}}_{n}+{\bar{p}}_{n}\right)\\ \text{ s.t. }\left(3.2\right)-\left(3.5\right)\\ \;m_{n}{\underline{p}}_{n}\geq {\underline{z}}_{n}\;\forall n\in N\\ \;M_{n}{\bar{p}}_{n}\geq {\bar{z}}_{n}\;\forall n\in N\\ \;{\underline{p}}_{n},{\bar{p}}_{n}\in B\;\forall n\in N, \end{array}$$

where *m*_*n*_ and *M*_*n*_ are large coefficients (*Big-M Method*). Note here that if ${\underline{z}}_{n}$ > 0 (${\bar{z}}_{n}>0$), ${\underline{p}}_{n}$ (${\bar{p}}_{n}$) is forced to be one, and will be minimized to be 0 otherwise. Although a safe choice can be made for *m*_*n*_ = ${\underline{b}}_{n}$, choosing a sufficiently big number for *M*_*n*_ is less straightforward. Depending on user preference, a possible safe choice for *M*_*n*_ could be 2–3× the maximum intake. Again, weights can be included to indicate the relative importance of a nutrient.

### 3.2. Computing the trade-off between costs and nutritional adequacy

The original cost objective, which was not included in the previous section, will be re-introduced here. As we now have two objectives over which we want to optimize, we are dealing with a bi-objective optimization problem. In general for a multi-criteria optimization it holds that one cannot minimize all objectives to their individual minima^1^. In other words, there are trade-offs between the objectives, which is especially challenging when they are conflicting. In the bi-objective LDOP this means that lowering the cost of a given solution is only possible while worsening the nutritional adequacy of the diet. Similar, one cannot improve the nutritional adequacy without raising the cost. In particular, it is not evident to what extent improving one of the objectives will worsen the other objective. This problem can be solved by finding all possible trade-offs between cost and nutritional adequacy.

Given a maximum allowed cost it is possible to determine the diet that achieves minimum nutritional inadequacy without exceeding the maximum cost. This can be modeled by including a cost constraint (15), while minimizing the nutritional inadequacy. Changing the maximum cost in this constraint allows to obtain different combinations of cost vs. nutritional adequacy. This procedure, commonly known as the ε-constraint method (19), does not guarantee to find all possible trade-offs unless the increments in the maximum cost are sufficiently small: it is simply impossible to know upfront what sufficiently small entails, unless all trade-offs are already known. Furthermore, this procedure can lead to “inefficient” or infeasible solutions. That is, if the chosen maximum cost is too low, it might be impossible to create a diet that meets the remaining non-relaxed constraints such as the exact energy constraint (infeasible). On the other hand, for a given cost there may exist a less expensive diet with the same nutritional content as was found by the LP^2^ (inefficient). This last consideration brings us to an understanding of solutions which are (in some sense) better than others: a solution is considered “good” or *efficient* if there exists no solution which performs strictly better in one objective without worsening the other.

Finding all these possible efficient trade-offs ensures that well-considered choices can be made without needing an a priori preference for one of the objectives and it helps to make decisions when a limiting budget is present, or when a nutritionally adequate diet is unaffordable or unattainable. Before we introduce a method that can find all possible trade-offs, we first have to formalize some concepts regarding efficient solutions (25). For ease of notation, let $C\left(x\right)=\underset{i\in I}{\sum }c_{i}x_{i}$ be the cost of a diet and let *Z*(*x*) be a measure for the nutritional inadequacy as defined by one of the achievement functions in Section 3.1, where *x* represents all decision variables in the model^3^. A solution *x* is then called *efficient* if there is no other diet which performs strictly better in one objective without worsening the other. The corresponding solution, or *criterion value*, *y* = (*C*(*x*), *Z*(*x*)) is referred to as a *nondominated point*.

**Figure 1:**
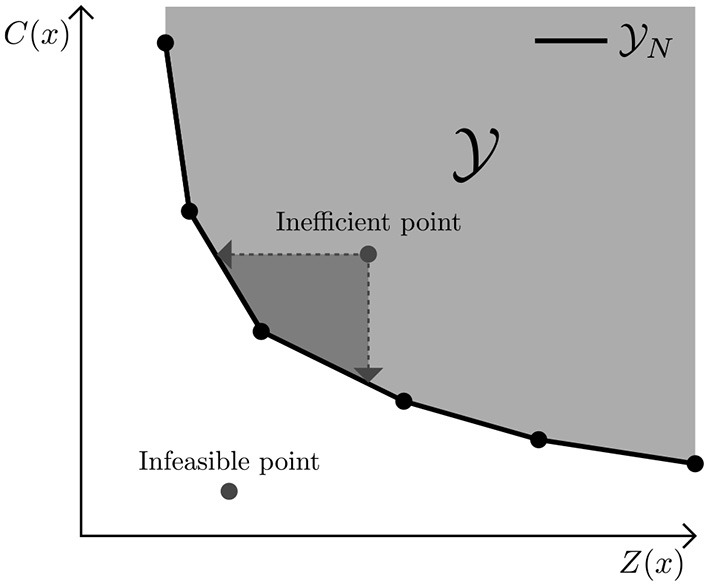
Example of a Pareto Frontier, where *C*(*x*) represents the cost of a diet and *Z*(*x*) the nutritional inadequacy. Y is the criterion space, which represents all feasible combinations of *C*(*x*) and *Z*(*x*). Y_*N*_ only contains nondominated points.

The set consisting of all feasible solutions, referred to as *decision space*, is often denoted with X. Here, *x* ∈ X consists of all possible values of the decision variables within the model. The *criterion space* Y consists of all feasible combinations of *C*(*x*) and *Z*(*x*) such that *x* ∈ X. The set containing all efficient solutions is known as X_*E*_ and the set of nondominated points as Y_*N*_^4^. Plotting all nondominated points generates a *Pareto curve*. In Figure 1 an example of a Pareto curve for a minimization problem is shown, where both objectives are linear. Note that an inefficient point can be improved in both objectives.

In the case of two linear objectives for which we can always find a feasible solution^5^, we can obtain all nondominated points using *weighted sum scalarization* (25). This technique gives a non-negative weight to each objective to indicate their relative importance. The combined objective can be written as a convex combination (non-negative weights summing up to 1) of *C*(*x*) and *Z*(*x*), that is


(3.6) $$\begin{array}{l} \min\lambda C\left(x\right)+\left(1-\lambda \right)Z\left(x\right) \end{array}$$

where λ ∈ [0, 1] is a weight which indicates the importance of *C*(*x*) relative to *Z*(*x*). Here, the required constraints depend on the chosen achievement function and the added dietary constraints. Note that the weighted sum scalarization only works for linear objectives with continuous variables and is thus suitable for the summed deviation and MinMax achievement function of Section 3.1, but not for the number of unmet constraints.

For a given λ ∈ (0, 1) the obtained solution *x* of Equation (3.6) is efficient and its corresponding criterion value nondominated. One could potentially solve for different values of λ to obtain efficient solutions and plot the corresponding nondominated points. In case one solves for λ = 0 or λ = 1, i.e., including only *C*(*x*) or *Z*(*x*), the solution can be *weakly efficient*. For λ = 0 this means that there are multiple possible diets which result in the same minimal nutritional inadequacy but have a different cost. We are of course only interested in the diet with the lowest cost, which is again an efficient point. This weak efficiency is resolved by using a *lexicographic* ordering. Here, we first optimize over one objective, after which we add the obtained objective value as a constraint and optimize over the other objective. For example, for λ = 0 we first optimize over the achievement function *Z* and obtain the objective value *Z*(x^*^) where x^*^ is our optimal solution. We then add the constraint *Z*(*x*) ≤ *Z*(x^*^) to the model and optimize over *C*.

**Figure 2:**
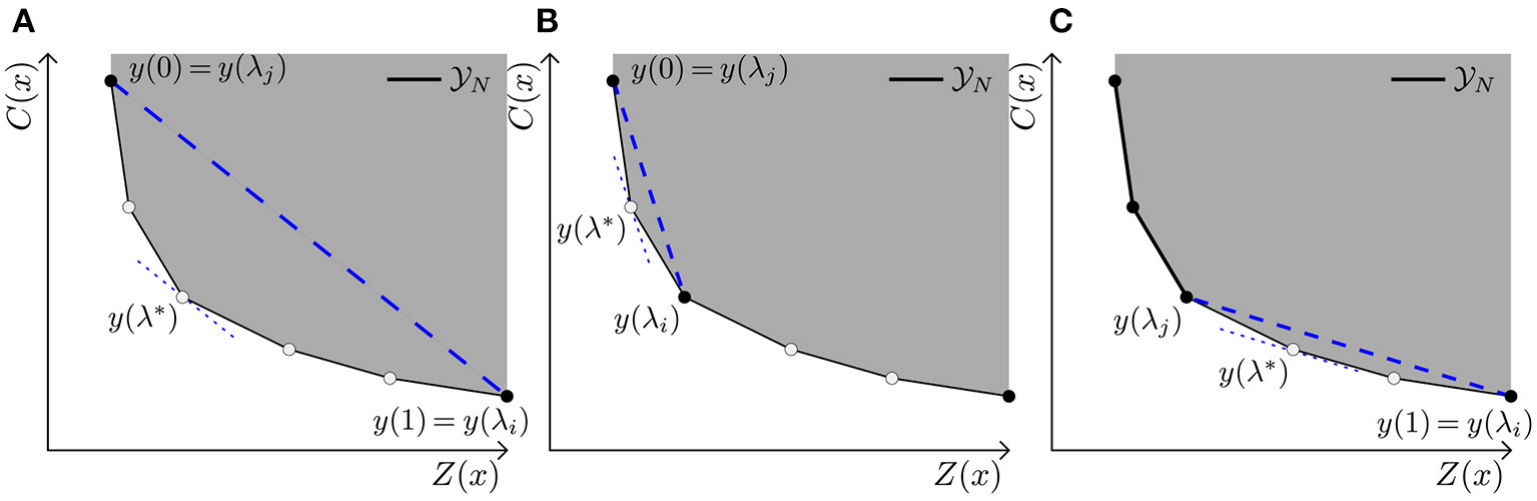
Representation of NISE with linear inequalities. As explained in Algorithm 1, the slope of already found efficient points is used to determine new efficient points. Here, when we refer to a “Line” we refer to a specific Line in Algorithm 1. **(A)** Based on the start solutions found in Line 3, a λ related to their slope is determined in Line 7. With λ = λ^*^ a new efficient point is found in Line 8. **(B)** Based on the found point, the area is split in $\left(y\left(\lambda_{i}\right),y\left(\lambda^{*}\right)\right)$ and $\left(y\left(\lambda^{*}\right),y\left(\lambda_{j}\right)\right)$, hence Line 13, and for both sides the same procedure can be repeated. That is, a new λ is determined resulting in a new efficient point. **(C)** Procedure is repeated until no new points are added to the existing Y_*N*_ approximation.

Solving for some set of λs does not necessarily result in the complete set Y_*N*_. Furthermore, as nicely stated by Das (41) “*an even spread of weights [λ] does not produce an even spread of points on the Pareto curve*.” Instead, it is possible to obtain the entire set Y_*N*_ using the NISE method (27), as we are dealing with a bi-objective LP. The NISE method is able to obtain the entire Pareto curve while only solving for “smart” choices of λ, i.e., λs that result in vertices/corner points of the Y_*N*_ front. In short, NISE uses the slope between already found efficient points to obtain a new λ which results in new efficient points as represented in Figure 2. These efficient points are either guaranteed to be vertices, or a linear combination of two other vertices. In case the latter happens, one has obtained a guarantee that there is no corner point in between the two earlier found corner points and can simply remove these non-corner points at the end.

In Algorithm 1 a method is described to find the complete Y_*N*_ front based on the NISE principles. Let *x*(λ) denote the optimal solution for a given λ. The corresponding non-dominated point is then *y*(λ) = (*C*(*x*(λ)), *Z*(*x*(λ))), which for ease of notation we denote with *y*(λ) = (*p*(λ), *q*(λ)).

**Algorithm 1:**
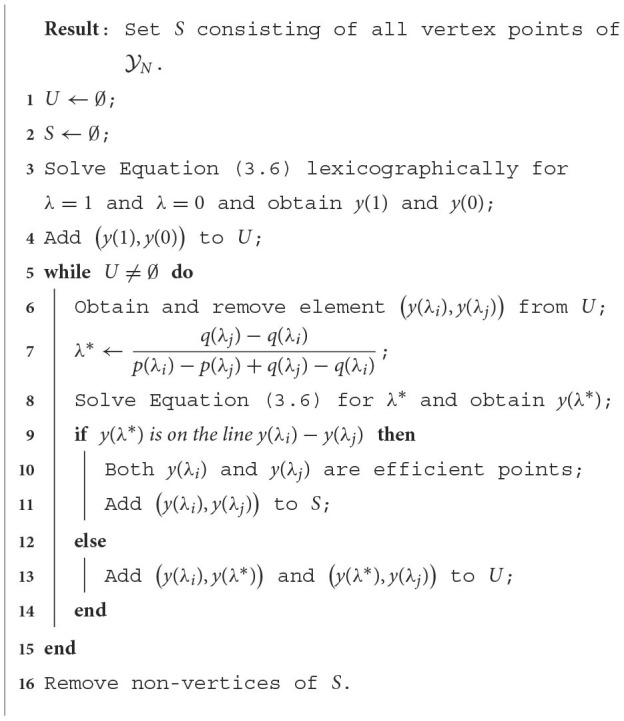
Obtain Y_*N*_ front (see Figure 2).

Solving Equation (3.6) in Line 3 always results in vertices in X_*E*_ and in Y_*N*_. However, Line 8 may result in a non-vertex non-dominated point, which can happen when the new found point is a linear combination of two corner points. Loosely speaking, this happens when the blue dashed line is parallel to an edge of the Y_*N*_ front as portrayed in Figure 2. Thus, in order to have non-dominated vertices only, the non-vertices have to be removed from *S* in Line 16.

**Figure 3:**
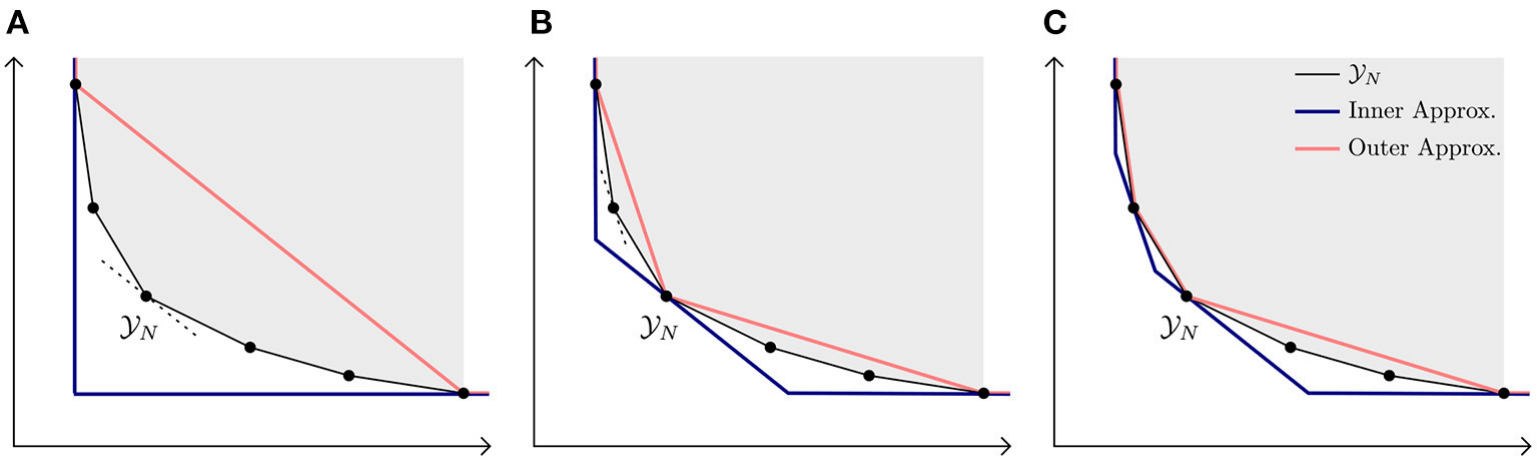
Obtaining inner and outer approximations (approximately). **(A)** Initial approximations based on the starting points. **(B)** After one iteration, the approximations are improved. **(C)** Approximations are further improved in second iteration.

There are some great advantages of using this procedure to determine Y_*N*_:

1. As mentioned in (42), it is easy to integrate this procedure into an existing LP solver, as the method iteratively calls the LP solver;
2. An alternative method to obtain the complete Y_*N*_ front uses simplex base changes (25). Our described procedure, however, is less prone to numerical issues as we do not heavily rely on solving and mutating linear inequalities;
3. As already stated, a well-known bi-objective approach is the ε-constraint method (19), where one of the two objectives is added as a constraint. In each iteration this constraint is further tightened to observe the impact on the (sole) objective. This, however, does not guarantee to find the complete Y_*N*_ front, unless ε is sufficiently small which normally results in many more calls to the LP solver than our described procedure;
4. The worst-case performance of this procedure is linear in the number of vertices *k* of Y_*N*_, that is a maximum of 4*k* − 5 LPs have to be solved for *k* ≥ 3. In Supplementary material Section 1 we briefly explain its interpretation;
5. The NISE algorithm can be extended in various ways in case the available solution time is limited (43), for example by limiting the solving time or the number of LPs it can solve, or by solving multiple LPs in parallel. It is also possible to obtain outer and inner approximations of Y_*N*_, which give insights in how the points found so far might deviate from the full set of non-dominated points. An example of obtaining these approximations is shown in Figure 3.

## 4. Analyses

In this section we present two applications in which the presented bi-objective approach can be used to analyze nutritional trade-offs in a dietary context. In Section 4.1 all efficient trade-offs between the cost and the nutritional adequacy of a diet are obtained. Based on the analysis we show the relevant insights that DMs can gain. In Section 4.2 the bi-objective approach is used to investigate how changes in energy intake affect the cost of a diet. This is done by only relaxing the exact energy intake and keeping all other nutrients within their bounds. Here, we show that a counterintuitive result where the cost of a diet decreases when increasing the energy intake can occur.

### 4.1. Relevant insights of trade-off costs vs. nutritional adequacy

In this section we describe an example where the bi-objective optimization procedure is used to obtain all efficient trade-offs between the cost and the nutritional adequacy of a diet. Based on this example we show several visualizations that can be acquired and how they benefit a DM. The summed deviation is selected as our achievement function as it is a linear objective, where the intake requirements for all nutrients are relaxed except the energy intake. Additionally, non-relaxed food commodity constraints are included. In the Supplementary material Sections 2, 3 a similar analysis for MinMax and the number of unmet constraints is reported. This leads to the following model:


$$\begin{array}{l} \min\lambda \sum_{i\in ℐ}c_{i}x_{i}+\left(1-\lambda \right)\sum_{n\in N}\left(\frac{{\underline{z}}_{n}}{{\underline{b}}_{n}}+\frac{{\bar{z}}_{n}}{{\bar{b}}_{n}}\right)\\ \text{ s.t. }\left(2.5\right)-\left(2.6\right)\\ \;\left(3.2\right)-\left(3.5\right)\\ \;\sum_{i\in ℐ}e_{i}x_{i}=E, \end{array}$$

where *e*_*i*_ denotes the energy content of food commodity *i* ∈ I and *E* the estimated average requirement (EAR). A new value for λ is determined in each iteration of Algorithm 1.

The list of available food commodities and food prices is obtained for the region Ebonyi, Nigeria^6^. We are particularly interested in the diet of a young adolescent female of 16–17 years, as it is often more challenging to meet her nutritional requirements than those of a male. This is because adolescent females need in general more iron than adolescent males of the same age while requiring less energy (44). Table 1 reports the nutritional requirements of the female, Table 2 contains the available food commodities in Ebonyi and their corresponding nutritional values and Table 3 states the cost per 100g and the reasonable minimum and maximum daily intake of the adolescent female for each food commodity in grams. We consider four diet types: omnivore, pescatarian, vegetarian and vegan. Here, the number of available food commodities decreases over the aforementioned diet types. We refer to a diet with fewer available food commodities as a more restrictive diet, e.g., vegan is more restrictive than pescatarian.

**Table 1:** Daily nutritional requirements of an adolescent male and female.

	**Source**	**Requirements**	
		**Female of 16–17 years**	**Male of 16–17 years**
Energy (kcal)	(45)	2, 503	3, 322
Protein (g)	(46)	≥46.34	≥54.2
Fat (g)	(47)	69.53–97.34	92.28–129.19
Calcium (mg)	(1, 48)	1,300–2,603	1,300–2,603
FolicAcid (Âμg DFE)	(1)	≥400	≥400
Iron Absorbed (mg)	(1, 49)	≥3.1	≥1.88
Iron (mg)	^**^	≤120	≤120
Magnesium (mg)	(1)	≥220	≥230
Niacin (mg NE)	(1, 50)	16–607	16–607
PantothenicAcid (mg)	(1)	≥5	≥5
Vitamin A (Âμg RE)	(1, 49)	600–2,637	600–2,637
Vitamin B1 (mg)	(1)	≥1.1	≥1.2
Vitamin B2 (mg)	(1)	≥1.0	≥1.3
Vitamin B6 (mg)	(1)	≥1.2	≥1.3
Vitamin B12(Âμg)	(1)	≥2.4	≥2.4
Vitamin C (mg)	(1, 48)	40–868	40–868
Zinc (mg)	(1)	≥7.2	≥8.6

The range reflects the recommended minimum intake and the upper limit of each nutrient.

In case only a lower or an upper limit is recommended, this is indicated with ≤ or ≥, respectively. Only the energy intake is assumed to be exact. Note that we do not model an upper limit on zinc, as in the time Cost of the Diet^*^ was compiled there was no evidence yet which linked excess intake of zinc to harmful effects. ^*^Cost of the Diet is a software tool conceptualized and developed by Save the Children, from which the list of requirements was obtained. For more information we refer the reader to Deptford et al. (9, 51).

** Based on conversations with the World Food Programme, we decided to raise the upper limit from 45 mg (49) to 120 mg to allow for both dietary and supplemental intake of iron.

**Table 2:** Nutritional properties reported per 100 g of food commodities available in Ebonyi.

**Name**	**Source**	**Energy**	**Protein**	**Fat**	**Ca**	**FA**	**Iron Abs**.	**Iron**	**Mg**	**Niacin**	**PA**	**Vit. A**	**Vit. B1**	**Vit. B2**	**Vit. B6**	**Vit. B12**	**Vit. C**	**Zinc**
		**(kcal)**	**(g)**	**(g)**	**(mg)**	**(Âμg DFE)**	**(mg)**	**(mg)**	**(mg)**	**(mg NE)**	**(mg)**	**(Âμg RE)**	**(mg)**	**(mg)**	**(mg)**	**(Âμg)**	**(mg)**	**(mg)**
Bambara groundnut, dried, raw	(52)	376	20.06	5.87	65.32	0	0.16	3.3	199.38	1.9	0.82	1.67	0.38	0.12	0	0	0	3.38
Bean, white, dried	(52)	335	22.13	1.48	73.98	395	0.29	5.72	185.84	1.64	0.5	14.92	0.89	0.11	0.51	0	0	3.77
Beans, green, raw	(52)	40	3.1	0.25	47.17	61.67	0.05	1.09	25	0.7	0.45	23.85	0.1	0.1	0.2	0	17.5	0.27
Beets, raw	(53)	43	1.61	0.17	16	109	0.04	0.8	23	0.33	0.16	2	0.03	0.04	0.07	0	4.9	0.35
Cabbage, raw	(52)	28	1.58	0.1	41	48.39	0.03	0.55	12	0.33	0.45	8.29	0.05	0.04	0.1	0	54	0.2
Carrot, raw	(52)	35	0.95	0.28	35	31.4	0.03	0.7	12.33	0.67	0.29	713.33	0.06	0.05	0.23	0	7	0.26
Chicken, clean, ready to cook	CotD^*^	143	13.5	9.5	7	3	0.18	0.7	10	5.42	0.38	20	0.05	0.12	0.13	0.12	0	0.9
Cocoyam, tuber, raw	(52)	129	2.4	0.2	10.64	22	0.03	0.6	13.15	0.8	0.37	0	0.1	0.03	0.24	0	8	0.38
Cowpea, black, dried, raw	(52)	301	21.08	1.6	69.28	412.29	0.28	5.54	202.07	3.03	0.5	0	0.7	0.15	0.35	0	0.79	3.89
Cowpea, brown, dried, raw	(52)	318	21.22	1.65	75.52	413.15	0.43	8.67	202	3.04	0.5	0	0.7	0.15	0.35	0	0.79	4.37
Cucumber, raw	(52)	15	0.7	0.1	13	6	0.02	0.45	12	0.3	0.45	2.92	0.02	0.01	0.04	0	14	0.17
Dikanut, kernel, dried, raw	(52)	704	7.9	66.87	164	0	0.17	3.4	0	0.7	0.82	0	0.18	0.09	0	0	0	0.28
Egg, chicken, raw	(52)	139	12.6	9.5	56	47	0.45	1.8	12	0.08	1.4	160	0.04	0.46	0.17	0.9	0	1.29
Eggplant, white, raw	CotD^*^	28	0.8	0.2	6	14	0.02	0.4	13	0.73	0.08	3	0.08	0.02	0.09	0	1	0.2
Fish, cod, atlantic, raw	(53)	82	17.81	0.67	16	7	0.1	0.38	32	2.06	0.15	12	0.08	0.06	0.24	0.91	1	0.45
Fish, dried, CotD	CotD^*^	290.32	56.56	5.44	647.8	28.32	0.45	1.8	115.52	17.66	0.83	5.52	0.11	0.18	0.59	6.27	0.24	2.92
Fish, mackerel, raw	(52)	124	19.7	5.03	28	1	0.2	0.8	33	5.3	0.6	39	0.14	0.14	0.4	2.4	0	0.49
Fish, tilapia, raw	(52)	99	18.8	2.7	17	24	0.32	1.3	36	3.5	0.6	26	0.04	0.06	0.24	1.58	0	0.83
Goat, feet	CotD^*^	70	6.5	4.7	1	2	0.1	0.4	6	2.02	0.1	0	0.02	0.04	0.09	0.49	0	1.1
Goat, meat, raw	(52)	165	17.5	10.58	10.75	5	0.59	2.37	27	6.12	0.55	0	0.18	0.29	0.4	1.13	0	3.45
Groundnut, shelled, dried, raw	(52)	578	22.4	45.87	46.68	110	0.2	3.9	190.91	15.46	0.82	0	0.87	0.14	0.59	0	0	2.52
Guava, fruit	(52)	57	1	0.41	23	7	0.04	0.7	13	1.22	0.18	35	0.05	0.04	0.14	0	261	0.32
Lamb, liver, raw	(52)	131	20.18	4.5	9	230	2.17	8.7	19	13	1.84	4970	0.32	3.07	0.9	90.1	4	4.66
Leaf, amaranth, raw	(52)	39	3.8	0.27	380	79	0.31	6.2	93	0.94	0.16	240.72	0.04	0.33	0.19	0	45	0.72
Leaf, eggplant, raw	(52)	45	4.38	0.74	331.5	118	0.22	4.3	58	1.35	0.16	295.74	0.15	0.4	0.3	0	79	0.73
Leaf, roselle, raw	(52)	40	2.81	0.24	212.33	117	0.2	4.1	58	1.2	0.16	215.32	0.17	0.45	0.3	0	33	0.9
Macaroni, dried	(52)	359	12.47	1.51	23	17	0.06	1.15	53.26	1.2	0.39	0	0.16	0.03	0.13	0	0	1.41
Maize, white, whole kernel, dried, raw	(52)	349	9.15	4.11	18.73	26	0.15	3.06	81.84	2.05	0.56	0	0.35	0.1	0.2	0	0	1.55
Maize, yellow, whole kernel, dried, raw	CotD^*^	353	9.04	4.45	12.37	26	0.18	3.54	120.5	2.2	0.56	50	0.33	0.15	0.4	0	0	1.7
Melon, seeds, slightly salted, raw	(52)	593	27.47	47.93	111.5	58	0.31	6.13	510	2.83	0.42	0	0.1	0.12	0.09	0	0	7.12
Milk, powder, fortified	CotD^*^	490	26.3	2.6	0	0	0	0	0	0.6	0	35	0.3	1	0	0	10	0
Millet, pearl, whole grain, raw	(52)	364	8.8	5.8	13.57	29.53	0.38	7.6	97.37	2.4	0.45	0.27	0.32	0.27	0.74	0	0	2.83
Mushroom, CotD	CotD^*^	27	2.2	0.5	6	18	0.08	1.7	12	5.4	2.16	0	0.07	0.3	0.1	0	4	0.9
Noodle, dried	CotD^*^	325	9.6	6.4	14	14	0.05	1	36	2.83	0.22	0	0.04	0.04	0.08	0	0	1
Oats	(53)	389	16.89	6.9	54	56	0.24	4.72	177	0.96	1.35	0	0.76	0.14	0.12	0	0	3.97
Oil, groundnut	(52)	900	0	100	0	0	0	0.03	0	0	0.01	0	0	0	0	0	0	0
Oil, palm, red	(52)	900	0	100	0	0	0	0.01	0	0	0.01	5720	0	0	0	0	0	0
Okra, raw	(52)	33	1.7	0.24	83.65	88	0.04	0.82	13	0.7	0.45	26.08	0.04	0.08	0.22	0	27.77	0.6
Onion, red	CotD^*^	44	1.4	0.2	22	15	0.01	0.2	11	0.53	0.11	0	0.04	0.02	0.13	0	5	0.2
Palm nuts, pulp	(52)	527	1.83	50.67	53.33	0	0.24	4.83	0	1.4	0.18	0	0.2	0.1	0	0	12	0
Peanut, with shell	CotD^*^	414	18.8	35.9	67	92	0.17	3.4	123	13.45	1.01	0	0.18	0.08	0.19	0	0	2.4
Peas, raw	CotD^*^	91	6.95	0.35	43.08	65	0.08	1.56	47.12	2.82	0.45	37.88	0.4	0.14	0.17	0	7.64	1.24
Pepper, sweet, red, raw	(52)	33	1.3	0.3	12	45	0.02	0.4	12	1.16	0.29	191	0.07	0.09	0.32	0	161.2	0.33
Pineapple, pulp	(52)	54	0.44	0.24	20.4	12	0.03	0.51	11.67	0.15	0.18	5.12	0.07	0.03	0.09	0	29.75	0.11
Plantain, ripe, raw	(52)	140	1.2	0.25	7.33	22	0.04	0.9	37	0.67	0.45	43	0.07	0.05	0.3	0	18.4	0.12
Potato, raw	(52)	80	1.88	0.11	10.65	17.5	0.04	0.87	27.33	1.22	0.37	1.13	0.08	0.12	0.27	0	17.25	0.34
Pumpkin, squash, raw	(52)	29	1	0.1	19.33	8	0.06	1.2	14	0.5	0.29	100	0.05	0.02	0.1	0	8	0.32
Rice, white, long grain, parboiled, unenriched, dry	(53)	374	7.51	1.03	71	8	0.04	0.74	27	5.05	0.67	0	0.22	0.05	0.45	0	0	1.02
Rice, white, raw	(52)	349	6.85	0.6	12	20	0.07	1.4	35	1.3	1.08	0	0.07	0.04	0.2	0	0	1.16
Sesame, seeds, whole, dried, raw	(52)	577	18.2	48.9	983	97	0.59	11.8	351	3.4	0.42	2.5	0.68	0.19	0.79	0	0	7.75
Sheep, tripe	CotD^*^	83	14.5	2.4	3	4	0.25	1	14	4.62	0.23	0	0.05	0.09	0.19	1.09	0	2.4
Shrimp, dried	CotD^*^	306	66.4	2.5	41	35	0.35	1.4	122	19.72	0.47	0	0.1	0.11	0.82	3.05	0	1.7
Sorghum, whole grain, raw	(52)	344	10.5	3.33	24.11	29.29	0.18	3.7	310.59	3.3	0.73	1.39	0.36	0.16	0.25	0	0	1.79
Soybean, dried, raw	(52)	410	31.97	16.98	231.56	375	0.39	7.77	245.26	2	0.5	1	0.7	0.28	0.82	0	0	4.73
Spaghetti, dry, unenriched	(53)	371	13.04	1.51	21	18	0.06	1.3	53	1.7	0.43	0	0.09	0.06	0.14	0	0	1.41
Sweet potato, pale yellow, raw	(52)	115	1.45	0.18	27.33	52	0.06	1.1	16	0.63	0.37	3.21	0.09	0.04	0.27	0	22.33	0.38
Tapioca, pearl, dry	(52)	358	0.19	0.02	20	4	0.08	1.58	1	0	0.14	0	0	0	0.01	0	0	0.12
Tomato paste, concentrated	(52)	89	4.3	0.5	64.3	10	0.15	2.98	60	3.08	0.45	75.1	0.06	0.15	0.22	0	17.8	0.4
Tomato, red, ripe, raw	(52)	22	1.01	0.18	12.97	21.25	0.03	0.6	13	0.56	0.45	51.97	0.06	0.04	0.08	0	29.64	0.7
Tomato, sundried	(53)	258	14.11	2.97	110	68	0.45	9.09	194	9.05	2.09	44	0.53	0.49	0.33	0	39.2	1.99
Watermelon, fruit	(52)	29	0.52	0.2	7	3.83	0.01	0.25	9.55	0.14	0.17	41.88	0.04	0.04	0.07	0	7.2	0.1
Wheat, whole grain, raw	(52)	326	12.35	2.2	30.34	45	0.24	4.7	140	5.55	1.13	0.25	0.46	0.1	0.28	0	0	1.7

The nutrient abbreviations are: Ca for Calcium, FA for FolicAcid, Abs. for Absorbed, Mg for Magnesium, PA for PanthothenicAcid and Vit. for Vitamin.

* CotD stands for the Cost of the Diet, which is a software tool conceptualized and developed by Save the Children. For more information we refer the reader to Deptford et al. (9, 51).

**Table 3:** Cost per 100 g^*^ in Nigerian naira (NGN) and daily allowed intake in grams for food commodities available in Ebonyi.

	**Cost (NGN)**	**Diet type**	**Female of 16–17 years**		**Male of 16–17 years**	
			**Min (g)**	**Max (g)**	**Min (g)**	**Max (g)**
Bambara groundnut, dried, raw	69.41	VG	0	45	0	60
Bean, white, dried	78.26	VG	0	180	0	240
Beans, green, raw	115.89	VG	0	585	0	780
Beets, raw	310.70	VG	0	855	0	1,140
Cabbage, raw	40.90	VG	0	585	0	780
Carrot, raw	57.99	VG	0	900	0	1,200
Chicken, clean, ready to cook	227.60	OM	0	135	0	180
Cocoyam, tuber, raw	55.37	VG	0	540	0	720
Cowpea, black, dried, raw	40.30	VG	0	180	0	240
Cowpea, brown, dried, raw	94.02	VG	0	180	0	240
Cucumber, raw	15.91	VG	0	585	0	780
Dikanut, kernel, dried, raw	281.69	VG	0	45	0	60
Egg, chicken, raw	98.24	VE	0	360	0	480
Eggplant, white, raw	32.67	VG	0	585	0	780
Fish, cod, atlantic, raw	792.82	PC	0	225	0	300
Fish, dried, CotD	111.34	PC	0	225	0	300
Fish, mackerel, raw	128.80	PC	0	225	0	300
Fish, tilapia, raw	245.05	PC	0	225	0	300
Goat, feet	157.00	OM	0	135	0	180
Goat, meat, raw	325.68	OM	0	135	0	180
Groundnut, shelled, dried, raw	84.88	VG	0	45	0	60
Guava, fruit	20.62	VG	0	495	0	660
Lamb, liver, raw	40.48	OM	0	225	0	300
Leaf, amaranth, raw	10.73	VG	0	855	0	1,140
Leaf, eggplant, raw	23.74	VG	0	855	0	1,140
Leaf, roselle, raw	135.34	VG	0	855	0	1,140
Macaroni, dried	55.80	VG	0	540	0	720
Maize, white, whole kernel, dried, raw	48.13	VG	0	585	0	780
Maize, yellow, whole kernel, dried, raw	55.05	VG	0	585	0	780
Melon, seeds, slightly salted, raw	304.19	VG	0	90	0	120
Milk, powder, fortified	355.07	VE	0	117	0	156
Millet, pearl, whole grain, raw	37.10	VG	0	495	0	660
Mushroom, CotD	73.54	VG	0	585	0	780
Noodle, dried	100.00	VG	0	585	0	780
Oats	82.54	VG	0	585	0	780
Oil, groundnut	144.33	VG	0	90	0	120
Oil, palm, red	142.92	VG	0	90	0	120
Okra, raw	61.24	VG	0	585	0	780
Onion, red	45.45	VG	0	585	0	780
Palm nuts, pulp	92.05	VG	0	495	0	660
Peanut, with shell	107.15	VG	0	45	0	60
Peas, raw	363.21	VG	0	585	0	780
Pepper, sweet, red, raw	217.42	VG	0	855	0	1,140
Pineapple, pulp	187.04	VG	0	495	0	660
Plantain, ripe, raw	38.03	VG	0	585	0	780
Potato, raw	37.50	VG	0	540	0	720
Pumpkin, squash, raw	43.64	VG	0	900	0	1,200
Rice, white, long grain, parboiled, unenriched, dry	55.09	VG	0	540	0	720
Rice, white, raw	50.12	VG	0	540	0	720
Sesame, seeds, whole, dried, raw	96.12	VG	0	90	0	120
Sheep, tripe	180.00	OM	0	225	0	300
Shrimp, dried	466.29	PC	0	270	0	360
Sorghum, whole grain, raw	37.43	VG	0	540	0	720
Soybean, dried, raw	59.94	VG	0	180	0	240
Spaghetti, dry, unenriched	54.52	VG	0	540	0	720
Sweet potato, pale yellow, raw	23.57	VG	0	540	0	720
Tapioca, pearl, dry	62.01	VG	0	540	0	720
Tomato paste, concentrated	142.86	VG	0	9	0	12
Tomato, red, ripe, raw	64.34	VG	0	270	0	360
Tomato, sundried	135.75	VG	0	270	0	360
Watermelon, fruit	29.21	VG	0	270	0	360
Wheat, whole grain, raw	83.41	VG	0	495	0	660

The minimum (Min) and maximum (Max) daily intake are specified for an adolescent male and female in grams. For each food commodity it is specified if it is allowed within a diet type, which reflects the most restrictive diet in which the food can be included. The diet types are abbreviated as follows: OM for omnivore, PC for pescatarian, VE for vegetarian and VG for vegan. Note that the minimum intake for all specified food commodities is zero. For completeness we mention it explicitly in the table.

* Cost are obtained from the World Food Programme Nigeria country office.

**Figure 4:**
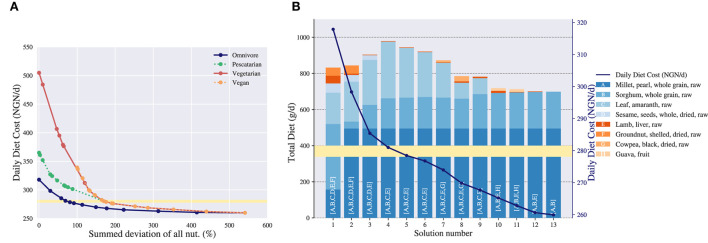
Trade-off analysis of the daily diet cost in Nigerian naira (NGN/d) vs. the nutritional deviation measured as summed deviation for an adolescent female of 16–17 years. in Ebonyi. **(A)** Pareto curve of the daily diet cost vs. the nutritional deviation. Four diet types are considered: omnivore, pescatarian, vegetarian and vegan. Each dot is an efficient solution representing a trade-off between cost and nutritional adequacy. The yellow highlighted area indicates a price level around 280 NGN. **(B)** Daily diet composition corresponding to the trade-offs of cost vs. nutritional deviation for the omnivore diet. The left *y*-axis shows the total amount of a food commodity included in a particular diet in grams and the right *y*-axis shows the cost of the diet that corresponds to the *y*-axis of **(A)**. The solutions in **(B)** correspond to the solutions of the omnivore diet in **(A)**. The food commodities included in a particular diet are indicated with letters on the corresponding bar, where the length of the bar indicates the total daily grams of a food commodity included in the diet. The yellow highlighted area indicates a price level around 280 NGN.

In Figure 4A the Pareto curves of all four diet types are shown. The *y*-axis represents the daily cost of a diet and the *x*-axis describes the summed deviation of all nutrients. Each dot is a corner point found in the bi-objective algorithm, and each point on the line can be translated to a particular diet with corresponding cost and summed deviation. The leftmost and rightmost dots are the starting points of the algorithm. The leftmost dot of each diet type represents the diet with the least summed deviation possible, whereas the rightmost dot represents the cheapest diet adhering to all non-relaxed constraints, i.e., the exact energy intake and the food commodity constraints.

Figure 4A allows for comparing diets on both a cost and a nutritional adequacy level. First of all, the leftmost dot on the curve shows whether it is possible to create a nutritious diet. In this example for all diet types except vegan a nutritious diet is possible, as the summed deviation is 0%. For the vegan diet, the leftmost dot has a summed deviation of 100%^7^, which indicates that a nutritious diet is not possible.

Second, the figure shows how the different diet types relate to each other. Here, the omnivore diet is overall the least expensive diet, as its Pareto curve is below the other curves. After the omnivore diet the most affordable diets are in order: pescatarian, vegetarian and vegan. This is a trivial result as each successive diet contains fewer food commodities than its predecessor, e.g., every vegan food commodity is vegetarian but not vice versa. In this case restricting food commodities results in more expensive diets, which indicates that the nutrients within these commodities are relevant for creating a both nutritious and affordable diet. Note that in general progressively excluding food commodities does not always lead to more expensive diets, as the excluded commodities might not be relevant when composing diets. Furthermore, we observe that the right-hand sides of the Pareto curves coincide when allowing for more nutritional deviation. This indicates that, in order to adhere to the exact energy intake and the food commodity constraints, only food commodities need to be included which are allowed in all diet types. Again, this is understandable as mostly foods that are good sources of energy are included (e.g., sorghum and millet) and these are not restricted within our chosen diet types.

Third, the curve shows what level of nutritional adequacy can be obtained when individuals have a restrictive budget. For example, if the female has a limited daily budget of 350 NGN, she can afford a nutritious omnivore diet with 0% deviation and 30 NGN left, a pescatarian diet with 10% deviation and 0 NGN left, a vegetarian diet with 85% deviation and 0 NGN left or a vegan diet with 100% deviation and 10 NGN left.

The Pareto curve gives an overview of all diet types together, but does not show the diet composition and nutritional content of an individual diet. Therefore, we show what additional information can be gained by investigating a diet type in more detail. For illustration purposes, we have selected the omnivore diet. As each point on the Pareto curve translates to a diet, we can pick the points on the curve and show their corresponding diet compositions. In Figure 4B a bar chart is presented which shows the diet composition of the omnivore diet given a certain cost. The *x*-axis represents the solution numbers, which are the vertices found from left to right in the Pareto curve, e.g., solutions 1 and 13 are the starting points. The left *y*-axis describes the cumulative number of gram included in a diet of a particular food commodity and the right *y*-axis shows the cost of a diet. The food commodities included in a solution are indicated with letters on the corresponding bar. Note that the right *y*-axis of Figure 4B translates to the *y*-axis of Figure 4A, and thus for each solution number its summed deviation is known as well.

Figure 4B shows that when allowing for more nutritional deviation, and thereby lowering the cost of the diet, the variety within the diet composition decreases. That is, fewer nutrient-dense foods are included, and the diet converges to a diet including only millet and sorghum, as they are good sources of energy. Furthermore, the figure shows the gradual change of the diet composition when allowing for more nutritional deviation. As an example, the “second-best” diet in terms of nutritional adequacy, solution 2, still contains the same food commodities as solution 1, though in different quantities.

**Figure 5:**
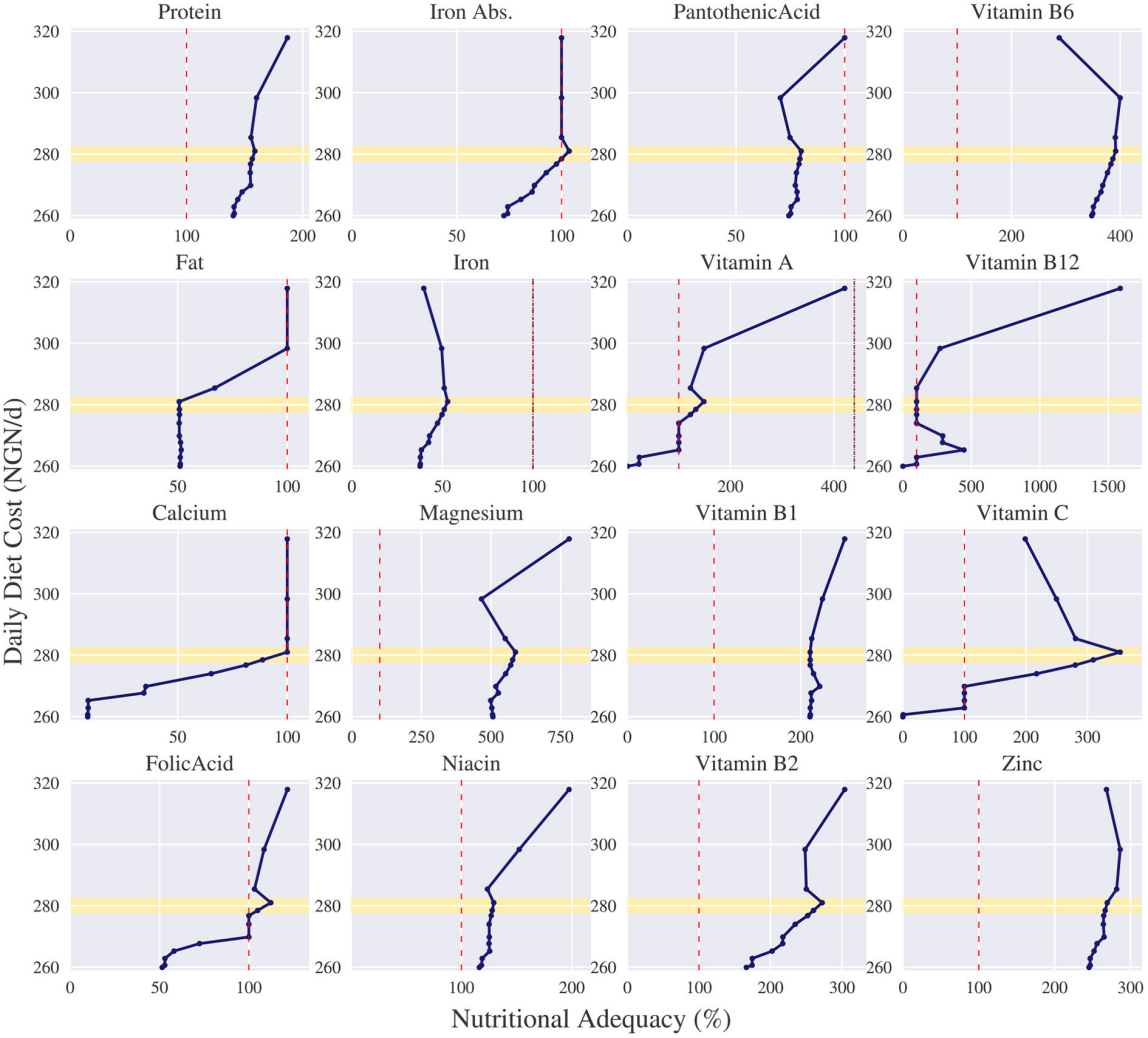
Nutritional adequacy per nutrient corresponding to the trade-offs of the daily diet cost in Nigerian naira (NGN/d) vs. nutritional deviation measured as summed deviation for the omnivore diet for an adolescent female of 16–17 years. in Ebonyi. For all nutrients, except iron, the adequacy is measured relative to their lower limit (100%). As iron only has an explicit upper limit, we measure its adequacy relative to its upper limit (100%). The daily cost of each dot corresponds to the daily cost on the Pareto curve. The lower limit of each nutrient is indicated with a red dotted line. If applicable, the upper limit is indicated with a dark red dotted line. The yellow highlighted area indicates a price level around 280 NGN.

For each solution we can also show the nutrient content of each nutrient w.r.t. its bounds. In Figure 5 the nutritional content for each nutrient is shown given a particular cost. The *x*-axis represents the nutritional adequacy of a given nutrient relative to its lower limit, 100%, which is indicated with a red dotted line. In case applicable, the upper limit is indicated with a dark red dotted line. Thus, all values above the 100% and below the dark red dotted line indicate nutritional adequacy of that nutrient. The *y*-axis describes the daily cost and corresponds with the cost in Figure 4A. The dots correspond to the corner points found on the Pareto curve.

Figure 5 shows which nutrients are problematic when faced with a restrictive budget. Before we found that with a maximum budget of 350 NGN, the female has no nutritional inadequacies if she chooses an omnivore diet. However, if her maximum budget is lowered to 280 NGN, which is highlighted in Figures 4, 5 with a yellow bar, then no nutritionally adequate diet can be composed. Figure 5 depicts that in that case the problematic nutrients in an omnivore diet are fat, calcium and panthothenic acid. Moreover, the figure shows that some nutrients are never problematic, such as protein, magnesium, niacin, vitamin B1, vitamin B2, vitamin B6 and zinc, as apparently enough of these nutrients are provided in the main sources of energy, sorghum and millet.

Concluding, this example shows how DMs can use a complete trade-off analysis to answer relevant questions, such as i) Is a nutritious diet possible given a certain diet type? If not, what are the nutritional deviations and costs of a “second-best” solution? ii) How does the level of nutritional inadequacy and the diet composition change when the budget changes? What level of nutritional adequacy is attainable when there is a strict budget? Which nutrients are problematic given this budget? iii) What are the differences in cost and nutritional adequacy between different diet types? How do these differences influence the diet composition and nutritional content of a diet?

### 4.2. Relaxing the energy constraint

Up until now we have regarded an exact energy intake, which is in line with current literature. In this section we question whether modeling the energy with an exact intake always makes sense. From an optimization point of view modeling an equality constraint is very restrictive and loosening it enables a larger feasible region, which gives more flexibility in finding solutions. Therefore, we use the bi-objective approach to show how the cost of a diet is affected by the energy intake. As in general using an equality constraint can result in the more-for-less paradox, we first have to explain in Section 4.2.1 what this paradox entails before we can understand the results. Several examples are illustrated in Section 4.2.2 to show the trade-offs between the energy intake and the cost of a diet and to demonstrate in which scenarios the aforementioned paradox may take place.

#### 4.2.1. More-for-less paradox energy intake

A common pitfall of having an equality constraint within an LP is the more-for-less paradox (54), where the minimization of a (seemingly!) more restrictive model is less costly than its less restrictive counterpart. This is aside from the fact that an equality constraint is very restrictive from an optimization standpoint, as all solutions are limited to a single plane in space. We illustrate this paradox by means of a toy example, where we consider two food commodities; item 1 is spinach and item 2 is flour which cost 40 and 3 cents per gram, respectively. Item 1 provides 0.25 kcal energy and 1.4 μg folic acid per gram, whereas item 2 provides 3.4 kcal energy and 0.3 μg folic acid per gram. The individual for whom we want to compose a diet is a female between 30 and 59 years with an energy requirement of 2,400 kcal. Additionally, a minimum of 400 μg of folic acid is required. These requirements translate to the following LP:


(4.1) $$\begin{array}{cc} \min & z=40x_{1}+3x_{2} \end{array}$$

(4.2) $$\begin{array}{cc} \text{s.t.} & \;0.25x_{1}+3.4x_{2}=2,400 \end{array}$$

(4.3) $$\begin{array}{c} \;1.4x_{1}+0.3x_{2}\geq 400 \end{array}$$

(4.4) $$\begin{array}{c} \;x_{1},x_{2}\geq 0. \end{array}$$

Here, the optimal solution is given by $x_{1}^{*}\approx 137$ g and $x_{2}^{*}\approx 696$ g with cost z^*^ ≈ 7, 552 cents. Equation (4.2) is binding by definition. Equation (4.3) is also binding, which indicates that the folic acid requirement is met exactly.

**Figure 6:**
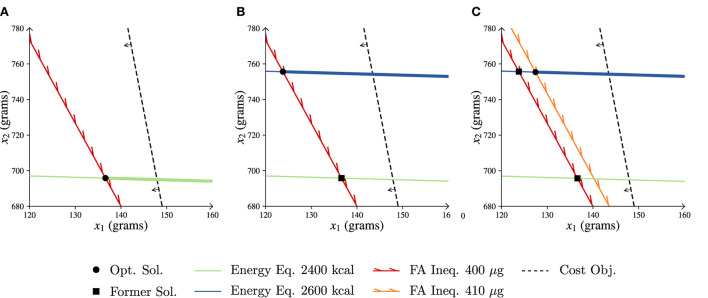
LP representations of the more-for-less paradox example. Opt. Sol. is optimal solution, former sol. is former solution, eq. is equality, ineq. is inequality, obj. is objective and FA folic acid. **(A)** Start situation. **(B)** Increased energy requirement. **(C)** Increased energy and FA requirements.

In Figure 6A a graphical representation of the feasible area is shown in which the optimal solution is highlighted. As there are only two commodities in our example, it is possible to illustrate the feasible area in a 2D-plane. On the *x*-axis the number of grams of item 1 are shown, whereas the *y*-axis represents the number of grams of item 2. The green solid line represents the energy equality constraint and shows all possible combinations of food commodities 1 and 2 that adhere to this equality. Similarly, the red solid line represents where the folic acid inequality is exactly the minimum required intake (400 μg). All possible combinations that result in a higher intake of folic acid are to the right side of this line. The further one moves to the right and upwards of the red line, the higher the folic acid intake of the diet will be as more of both commodities are added to the diet. So, the feasible region from which diets can be constructed is represented by the bold green line, as on this line both the energy constraint and the folic acid inequality hold. Now that we have identified the feasible area of this example, the optimal solution still has to be located. The black dashed line indicates the cost objective, and all combinations on this line result in the same cost for the diet. Moving to the left from the line reduces the cost, as less of each commodity is included. Thus, the optimal solution is at the leftmost part within the feasible area, which is at the intersection of the green and red solid lines.

We now assume that the female's energy intake is increased to 2,600 kcal. One would expect that a higher energy intake leads to higher costs, as the requirements increase. The increase in energy intake is graphically represented in Figure 6B by the blue solid line. Note that the position of the blue line has moved upward relatively to the green line. In order to adhere to the increased energy intake, more grams of *x*_2_ are required when keeping the amount of *x*_1_ fixed and vice versa. The feasible area has been completely altered and the previous optimal solution is no longer feasible. The black dashed line of the cost objective can move even further to the left, which represents a decrease in the optimal cost. The optimal solution is given by $x_{1}^{*}\approx 124$ g and $x_{2}^{*}\approx 756$ g with cost z^*^ ≈ 7, 219 cents, which is cheaper than the previous diet that had a lower energy intake.

Again, we adjust the female's requirements. On top of the increased energy intake, her required folic acid intake increases to 410 μg. The optimal solution is in this case given by $x_{1}^{*}\approx 131$ g and $x_{2}^{*}\approx 755$ g with cost z^*^ ≈ 7, 507 cents. In Figure 6C the increase in the folic acid requirement is represented by the orange solid line. Note that as the line is to the right of the red line (original folic acid requirement) the folic acid requirement has indeed increased. This decreases the size of the feasible region, and thus increases the cost with respect to the situation as presented in Figure 6B. However, this solution is still less expensive than the initial diet presented in Figure 6A. Thus, although all the requirements have strictly increased, this new optimal solution is still less expensive.

In the above example, while more nutrients are required the cost of the diet is lowered. This seems contradictory, as tightening the constraints usually results in a smaller feasible space and thus higher costs. The crucial aspect in this example, however, is that adjusting the equality constraint changes the feasible space instead of tightening it, which is shown in Figure 6B. This counterintuitive result is explained by the more-for-less paradox (54), where the right-hand side of an equality (or multiple equalities) can be increased while simultaneously improving on the objective function. Only under certain conditions related to the dual of this problem the paradox is induced. In Supplementary material Section 4 we provide a more in-depth explanation for the interested reader.

The counterintuitive result that occurred within our small example also occurs in larger datasets. We elaborate on two instances where the more-for-less paradox can take place. In the first instance the conclusions are straightforward and widely accepted, whereas the second instance may seem contradictory.

The first instance concerns the dietary requirements of adolescent females. For most micronutrients they require a higher intake, e.g., iron, than their male counterparts, while their energy intake is lower. Therefore, adolescent females require a relatively large amount of nutrient-dense foods in order to satisfy their nutritional requirements. In order to obtain the same amount of micronutrients with energy-dense foods as with nutrient-dense foods, one would have to consume many more calories. As energy-dense foods are usually less expensive than nutrient-dense foods, it is cheap to consume more calories to obtain the same amount of micronutrients. Thus, increasing the energy intake for adolescent females can reduce the cost of their diet, which corresponds to the more-for-less paradox.

The second instance extends the argument presented in our example and explains why the dietary cost of a non-pregnant female can be higher than those of a pregnant female of the same age and physique. During a pregnancy, a female requires strictly more energy, fat, protein and micronutrients than a non-pregnant female. Increasing the nutritional requirements normally tightens the feasible area and (potentially) increases the cost of a diet. In case the more-for-less paradox occurs, the increased energy intake induces a stronger decrease in cost than the increase in cost caused by the tightened nutritional requirements, hence the overall cost decreases.

#### 4.2.2. Bi-objective optimization for analyzing the effects of energy intake

In this section the bi-objective approach is used to detect whether the more-for-less paradox takes place, to find the minimum amount of calories needed to create a nutritious diet similar to one of the questions raised in (6), and to understand how the energy intake influences the cost of a nutritious diet. In our example two adolescent individuals of the Ebonyi region are considered: a male and female aged 16–17 years. Their respective nutritional requirements are reported in Table 1, where the nutritional requirements of the female relative to her energy requirement are high compared to those of the male. Here, we make use of the same data as provided in Section 4.1.

In our previous example all nutrients were included in the GP achievement function, while the energy constraint and other constraints were non-relaxed. This allowed us to obtain all trade-offs between cost and nutritional adequacy. In a similar fashion we now only relax the exact energy intake—and keep all other constraints non-relaxed—in order to obtain trade-offs between the energy intake and the cost of a diet. We relax the energy constraint such that it can have a negative and a positive deviation from its original intake. Note that the corresponding diets are nutritionally adequate; they only allow for an inadequacy in energy intake. This leads to the following model:


$$\begin{array}{l} \min\lambda \sum_{i\in ℐ}c_{i}x_{i}+\left(1-\lambda \right)\left(\frac{{\underline{z}}_{\text{energy}}}{E}+\frac{{\bar{z}}_{\text{energy}}}{E}\right)\\ \text{ s.t. }\left(2.2\right)-\left(2.6\right)\\ \;\sum_{i\in ℐ}e_{i}x_{i}=E-{\underline{z}}_{\text{energy}}\\ \;\sum_{i\in ℐ}e_{i}x_{i}=E+{\bar{z}}_{\text{energy}}\\ \;{\underline{z}}_{\text{energy}},{\bar{z}}_{\text{energy}}\geq 0 \end{array}$$

where ${\underline{z}}_{\text{energy}}$ and ${\bar{z}}_{\text{energy}}$ are decision variables that reflect the under- and overconsumption of energy, respectively, see Section 3.1. A new value for λ is determined in each iteration of Algorithm 1.

**Figure 7:**
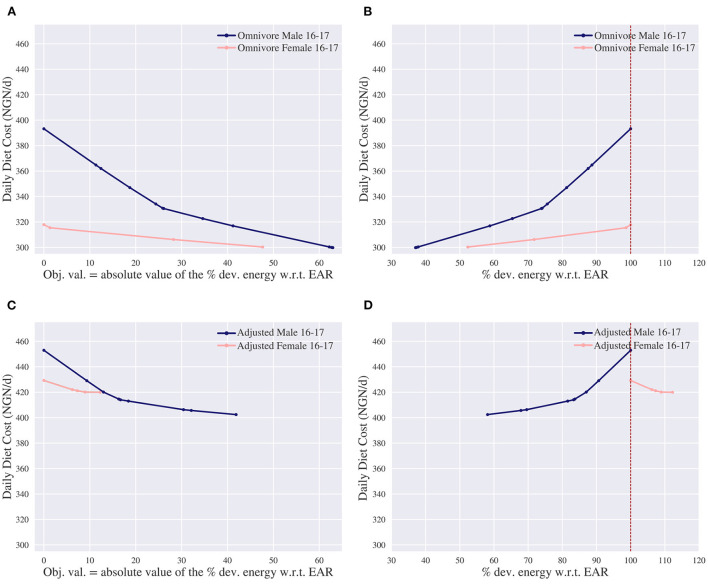
Trade-offs of the daily diet cost in Nigerian naira (NGN) vs. energy deviation (dev.) for the omnivore and adjusted omnivore diet. The adjusted omnivore considers the same food commodities as omnivore, but excludes “Lamb, liver, raw,” “Leaf, amaranth, raw” and “Sesame, seeds, whole, dried, raw.” EAR denotes the estimated average requirement. **(A)** Pareto curve for the omnivore diet of the daily diet cost vs. absolute value of the deviation of energy w.r.t. EAR. **(B)** Energy deviation w.r.t. EAR of the omnivore diet. This is different from **(A)**, as **(A)** shows the model's objective value which is the absolute value of the deviation in **(B)**. **(C)** Pareto curve for the adjusted omnivore diet of the daily diet cost vs. absolute value of the deviation of energy w.r.t. EAR. **(D)** Energy deviation w.r.t. EAR of the adjusted omnivore. This is different from **(C)**, as **(C)** shows the model's objective value which is the absolute value of the deviation in **(D)**.

We first consider an omnivore diet. In Figure 7A the trade-off is shown between cost and energy deviation for both individuals. Here, the leftmost solution of the female corresponds with the leftmost solution of the omnivore diet in Figure 4A, as the energy and nutritional inadequacy for both problems are minimized to 0. Figure 7B reports whether the energy deviation is below or above the EAR. This differs from Figure 7A, as Figure 7A shows the total deviation, i.e., the model's objective value, which is the absolute value of the deviation in Figure 7B. From these figures it is observed that it is still possible to create a nutritionally adequate diet with only 37% and 52% of the original required energy intake for the male and female, respectively. Apparently there are enough affordable nutrient-dense commodities available to create nutritionally adequate diets.

In these diets the more-for-less paradox does not take place: the cost decreases when the energy intake decreases. This seems in line with the previous observation about the availability of nutrient-dense commodities. That is, we argued that the more-for-less paradox most likely takes place when there is a lack of nutrient-dense foods and for the omnivore diet there seems to be enough nutrient-dense food. To further support this reasoning, a diet is considered where some nutrient-dense commodities are omitted. We exclude three nutrient-dense foods which were present in the nutrient and energy adequate diet of the female: “Lamb, liver, raw,” “Leaf, amaranth, raw” and “Sesame, seeds, whole, dried, raw”^8^. This diet is referred to as “adjusted-omnivore.”

Figure 7C shows the trade-off between cost and energy deviation for both individuals of the adjusted-omnivore diet and Figure 7D reports whether the deviation is above or below the EAR. For the male, it is possible to create a nutritionally adequate diet with less energy; at least 58% of the EAR is required. The more-for-less paradox does not take place, as more calories are needed to create a nutritionally adequate diet. For the female, it is more cost-effective to increase the energy intake up to 12% to lower the cost. In this case, the more-for-less paradox takes place. These observations are in line with our expectations as not only the adjusted-omnivore diet has fewer nutrient-dense commodities than the omnivore diet, but the female has higher nutritional requirements per kcal intake than the male as well.

## 5. Discussion

Finding the complete Pareto curve for two linear objectives has not been considered before in the current diet optimization literature. Finding all possible trade-offs is relevant as it accurately shows how the objectives impact each other. In the current literature the ε-constraint method is used to approximate the trade-off curve (19) for example to investigate the nutritional adequacy vs. cost (18, 55), cost vs. environmental impact (21) and environmental impact vs. deviations from the current dietary pattern (22–24). The ε-constraint method, however, does not guarantee to find the complete trade-off curve without solving many uninformative LPs. Therefore, we presented a bi-objective algorithm based on the NISE procedure (27) that is guaranteed to find all efficient trade-offs between any two linear objectives in an LP context without solving uninformative LPs, and of which the worst-case performance is linear in the number of vertices on the Pareto curve. For this algorithm no a priori preference is needed of the DM.

As an application for this algorithm we particularly focused on how to balance costs and nutritional adequacy in a dietary context. In our first analysis we modeled the nutritional inadequacy using GP with a summed deviation. We explained relevant insights that can be obtained for organizations such as the United Nations World Food Programme, as they make use of diet optimization to understand the cost of an affordable and nutritious diet (5). At the World Food Programme diet optimization is part of an analysis known as Fill the Nutrient Gap, which “*aims to support identification of strategies to increase availability, access, and choice of nutritious foods, to ultimately improve nutrient intake*” (5). Our analysis contributes to their current research practice, as the bi-objective algorithm provides insights in the relation between costs and nutritional adequacy of a diet.

The second analysis focused on the effects of relaxing the energy intake requirement while all other nutritional requirements had to be met. This analysis is an extension to (6) where the energy intake of a nutritious diet is minimized. We showed when the more-for-less paradox takes place (54). By understanding the paradox, one can better grasp seemingly counter-intuitive situations, e.g., a nutritious diet of a pregnant female is less expensive than that of a non-pregnant female despite the pregnant female having strictly higher nutritional needs. We argued that the more-for-less paradox is most likely to occur when insufficient affordable nutrient-dense foods are available. In our example of the Ebonyi region all considered food commodities are minimally processed or even unprocessed, which make them relatively nutrient-dense. However, in general in middle- and high-income countries there is a rise of ultra processed foods (56), which are less nutrient rich. Hence, we suspect that the more-for-less paradox is likely to occur in countries experiencing this nutrition transition. This emphasizes the relevance of this topic as it shows whether enough affordable nutrient-dense foods are available.

Within our main analyses we used the summed deviation as our GP achievement function to measure nutritional inadequacy. The main advantage of this measure is that it considers all nutrients simultaneously, and that the combined deviation determines the objective value. A disadvantage is that it is unknown beforehand how the summed deviation is distributed among the nutrients. That is, a summed deviation of 100% could indicate that all 15 nutrients are deviating with 6.7%, or that only one nutrient deviates with 100% while the others remain within their requirements. One might prefer the former over the latter situation. The MinMax achievement function can circumvent this problem as it minimizes the worst-case performance over all nutrients. The downside of the MinMax achievement function is that it is unknown beforehand to what extent all nutrients other than the worst performing nutrient deviate. For the interested reader we have included the trade-off analysis of cost vs. MinMax deviation in Supplementary material Section 2. The proposed algorithm can be applied to the MinMax objective function as this is a linear objective function. In order to combine the strengths of both achievement functions, one could also consider both achievement functions simultaneously (16). In this case a new objective is constructed that measures the nutritional inadequacy of a diet, where one has to specify the weights of the summed deviation and MinMax beforehand.

In our GP formulation it is possible that for some nutrients the amount of deficiency or excess is unacceptable from a health perspective. To avoid an undesired outcome where the nutrient intake is below or above a certain level, one can include additional constraints that require an absolute lower or upper limit (16). This can still lead to nutritional infeasibilities. A solution would be the use of piecewise linear penalties (48) where excessive deviations above the lower limit and upper limit are punished more severely within the objective. This is beyond the scope of our research.

Throughout the paper we mainly focused on the cost and the nutritional adequacy of a diet, but it is also possible to apply this algorithm to other relevant (linear) objectives. Recent literature overviews (57, 58) show the increased interest in and importance of including environmental aspects within diet optimization. Therefore, one could also consider the minimization of greenhouse gas emission, water footprint or land usage related to a diet. Another relevant application relates to the healthcare sector: (59) describes how to minimize the distance between an existing diet pattern and an advised pattern to make the diet more nutritious without rigorous diet adjustments in order to prevent cancer. Here, one could investigate the trade-off between nutritional adequacy and deviation from the existing diet pattern to help select a diet that is more nutritious and still acceptable for the patient.

The presented algorithm focuses on finding the trade-offs between two linear objectives. One might be interested in finding all trade-offs between more than two objectives, e.g., cost, nutritional adequacy, deviation from the current food pattern and environmental impact. Although it is possible to extend the algorithm to more than two dimensions, there are some important caveats. As explained in (60) using more than two dimensions can result in (partly) negative weights for the weighted sum method. Thus, not every solution is guaranteed to be (weakly) efficient, and many LPs will be solved that give solutions that are not on the trade-off curve. In the context of radiotherapy, (42) suggest an approximation technique where they construct a positive weight in case the actual weight is (partly) negative. This however does not guarantee that the complete trade-off curve is obtained. In our current research we are working toward a way to minimize the number of LPs that solve inefficient points, while still obtaining the complete trade-off curve.

Note that the overall purpose of this paper is to show how one can analyse the trade-offs between conflicting objectives in a dietary context and to illustrate what types of insights can be gained. We do not necessarily recommend the diets which are obtained when allowing some form of nutritional inadequacy during the trade-off analyses. Depending on the goal and preferences of the DM, different constraints can be added or nutrients can be included or excluded when measuring nutritional deviations.

## Data availability statement

The original contributions presented in the study are included in the article/Supplementary material, further inquiries can be directed to the corresponding author.

## Author contributions

MK was responsible for the overall research and writing. MB and HF had an advisory role throughout the research and helped to structure and revise the text. HF initiated the research. All authors contributed to the article and approved the submitted version.

## Footnotes

^1^Here, we assume that the *ideal point* is not attainable, which in our case is a diet which minimizes both the single cost objective and the single achievement function to their individual minima.

^2^This is referred to as a *weakly efficient* solution.

^3^As an example, for the summed deviation as described in Section 3.1.1, it holds that *x* has length |I| + 2|N|.

^4^This notation is not unique; an overview of different notations can be found in (25).

^5^See (25) for all conditions. In short, it is relevant that the decision space and the criterion space are both convex and closed, which holds in our case since we have linear functions and reasonable lower and upper limits for each food commodity. Furthermore, it should hold that the criterion space is bounded from below, which holds for the LDOP as the cost and the nutritional achievement function are both trivially bounded from below by 0.

^6^Food prices are obtained from the World Food Programme Nigeria country office.

^7^This is caused by a complete vitamin B12 deficiency, as there is no possibility to include vitamin B12 with the available food commodities.

^8^As the leftmost solution in Figure 4A equals the leftmost solution in Figure 7, their diet composition is the same, see Figure 4B.
