# Corrigendum: Bi-objective goal programming for balancing costs vs. nutritional adequacy

**DOI:** 10.3389/fnut.2023.1239915

**Published:** 2023-07-11

**Authors:** Melissa F. Koenen, Marleen Balvert, Hein Fleuren

**Affiliations:** ^1^Zero Hunger Lab, Tilburg School of Economics and Management, Tilburg University, Tilburg, Netherlands; ^2^Department of Econometrics and Operations Research, Tilburg School of Economics and Management, Tilburg University, Tilburg, Netherlands

**Keywords:** diet optimization, goal programming, bi-objective, nutritional adequacy, linear programming

In the published article, there was an error in **Table 3** as published. For some food commodities the entries were duplicated, whereas for other food commodities they were not present.

The corrected Table 3 and its caption appear below.

**Table 3:** Cost per 100 g^*^ in Nigerian naira (NGN) and daily allowed intake in grams for food commodities available in Ebonyi.

	**Cost (NGN)**	**Diet type**	**Female of 16–17 years**		**Male of 16–17 years**	
			**Min (g)**	**Max (g)**	**Min (g)**	**Max (g)**
Bambara groundnut, dried, raw	69.41	VG	0	45	0	60
Bean, white, dried	78.26	VG	0	180	0	240
Beans, green, raw	115.89	VG	0	585	0	780
Beets, raw	310.70	VG	0	855	0	1,140
Cabbage, raw	40.90	VG	0	585	0	780
Carrot, raw	57.99	VG	0	900	0	1,200
Chicken, clean, ready to cook	227.60	OM	0	135	0	180
Cocoyam, tuber, raw	55.37	VG	0	540	0	720
Cowpea, black, dried, raw	40.30	VG	0	180	0	240
Cowpea, brown, dried, raw	94.02	VG	0	180	0	240
Cucumber, raw	15.91	VG	0	585	0	780
Dikanut, kernel, dried, raw	281.69	VG	0	45	0	60
Egg, chicken, raw	98.24	VE	0	360	0	480
Eggplant, white, raw	32.67	VG	0	585	0	780
Fish, cod, atlantic, raw	792.82	PC	0	225	0	300
Fish, dried, CotD	111.34	PC	0	225	0	300
Fish, mackerel, raw	128.80	PC	0	225	0	300
Fish, tilapia, raw	245.05	PC	0	225	0	300
Goat, feet	157.00	OM	0	135	0	180
Goat, meat, raw	325.68	OM	0	135	0	180
Groundnut, shelled, dried, raw	84.88	VG	0	45	0	60
Guava, fruit	20.62	VG	0	495	0	660
Lamb, liver, raw	40.48	OM	0	225	0	300
Leaf, amaranth, raw	10.73	VG	0	855	0	1,140
Leaf, eggplant, raw	23.74	VG	0	855	0	1,140
Leaf, roselle, raw	135.34	VG	0	855	0	1,140
Macaroni, dried	55.80	VG	0	540	0	720
Maize, white, whole kernel, dried, raw	48.13	VG	0	585	0	780
Maize, yellow, whole kernel, dried, raw	55.05	VG	0	585	0	780
Melon, seeds, slightly salted, raw	304.19	VG	0	90	0	120
Milk, powder, fortified	355.07	VE	0	117	0	156
Millet, pearl, whole grain, raw	37.10	VG	0	495	0	660
Mushroom, CotD	73.54	VG	0	585	0	780
Noodle, dried	100.00	VG	0	585	0	780
Oats	82.54	VG	0	585	0	780
Oil, groundnut	144.33	VG	0	90	0	120
Oil, palm, red	142.92	VG	0	90	0	120
Okra, raw	61.24	VG	0	585	0	780
Onion, red	45.45	VG	0	585	0	780
Palm nuts, pulp	92.05	VG	0	495	0	660
Peanut, with shell	107.15	VG	0	45	0	60
Peas, raw	363.21	VG	0	585	0	780
Pepper, sweet, red, raw	217.42	VG	0	855	0	1,140
Pineapple, pulp	187.04	VG	0	495	0	660
Plantain, ripe, raw	38.03	VG	0	585	0	780
Potato, raw	37.50	VG	0	540	0	720
Pumpkin, squash, raw	43.64	VG	0	900	0	1,200
Rice, white, long grain, parboiled, unenriched, dry	55.09	VG	0	540	0	720
Rice, white, raw	50.12	VG	0	540	0	720
Sesame, seeds, whole, dried, raw	96.12	VG	0	90	0	120
Sheep, tripe	180.00	OM	0	225	0	300
Shrimp, dried	466.29	PC	0	270	0	360
Sorghum, whole grain, raw	37.43	VG	0	540	0	720
Soybean, dried, raw	59.94	VG	0	180	0	240
Spaghetti, dry, unenriched	54.52	VG	0	540	0	720
Sweet potato, pale yellow, raw	23.57	VG	0	540	0	720
Tapioca, pearl, dry	62.01	VG	0	540	0	720
Tomato paste, concentrated	142.86	VG	0	9	0	12
Tomato, red, ripe, raw	64.34	VG	0	270	0	360
Tomato, sundried	135.75	VG	0	270	0	360
Watermelon, fruit	29.21	VG	0	270	0	360
Wheat, whole grain, raw	83.41	VG	0	495	0	660

The minimum (Min) and maximum (Max) daily intake are specified for an adolescent male and female in grams. For each food commodity it is specified if it is allowed within a diet type, which reflects the most restrictive diet in which the food can be included. The diet types are abbreviated as follows: OM for omnivore, PC for pescatarian, VE for vegetarian and VG for vegan. Note that the minimum intake for all specified food commodities is zero. For completeness we mention it explicitly in the table.

^*^Cost are obtained from the World Food Programme Nigeria country office.

The authors apologize for this error and state that this does not change the scientific conclusions of the article in any way. The original article has been updated.

