# Supplementary material for: Bi-objective goal programming for balancing costs vs. nutritional adequacy
Source: Front Nutr. 2022 Dec 15;9:1056205. doi: 10.3389/fnut.2022.1056205 (PMC9798409; doi:10.3389/fnut.2022.1056205)
Supplement: Supplementary file 1 [file Data_Sheet_1.pdf]

Supplementary Material

1 WORST-CASE PERFORMANCE OF THE BI-OBJECTIVE OPTIMIZATION ALGORITHM

In Section 3.2 we mentioned some of the advantages of Algorithm 1 that determines the complete $\mathcal{Y}_N$ front for two linear objectives based on the NISE principles (27). One of its advantages is its computational performance, as the algorithm is linear in the number of vertices k of $\mathcal{Y}_N$. That is, a maximum of $4k-5$ LPs have to be solved when $k \geq 3$. In this section we briefly explain the intuition behind this worst-case performance and for the interested reader we provide a formal proof. They key insight is that one can break down the worst-case performance of a problem into smaller structures for which one already has determined its worst-case performance. Note that in the following we consider a minimization problem without loss of generalization.

As an example, let us consider $k = 5$ where we show that its substructures depend on $k = 3$ and $k = 4$. Initially 4 LPs are solved to obtain the starting points. The next efficient point that we find, by solving 1 LP using λ^* in Line 8, is not guaranteed to be a vertex point. Thus, we make a distinction between either ending up on a vertex and not ending up on a vertex. Since $k = 5$, we have three possible vertices which we can find. For sake of reference we refer to these three vertices as v_1, v_2 and v_3, where v_1 is to the 'left' of v_2 and v_2 to the 'left' of v_3, that is v_1 performs better in the first objective than v_2 and v_2 performs better in the first objective than v_3. Let us first assume we end up on a vertex. If we end up at v_1, then we need to solve 1 LP to find out that indeed no vertex is found to the left of v_1, Line 9, and to the right of v_1 is a substructure similar to $k = 4$, of which the starting points are found, while v_2 and v_3 remain to be found. If we end up at v_2, then at either side of v_2 we have a structure similar to $k = 3$, again for which the starting points are known. Ending up at v_3 results in the same but mirrored structure as v_1. Let us now assume we do not end up on a vertex. We can either end up on the edge between v_1 and v_2 or on the edge between v_2 and v_3. As these structures are similar we assume without loss of generality that we end up on a point between v_1 and v_2. Then, to the left of this point is v_1 and one of the starting points, which equals a substructure of $k = 3$ of which the starting points are known, and to the right of this point we have v_2, v_3 and the other starting point, which equals a substructure of $k = 4$ for which the starting points are known.

In the example above, all explained cases depend on the worst-case performance of the substructures with $k = 3$ and $k = 4$. Therefore, one can also determine their worst-case performance to consequently determine the worst-case performance of $k = 5$. In Figure S1 the worst-case performance for $k = 3, 4, 5$ is shown, where we show how the worst-case performance of $k = 4$ and $k = 5$ ultimately depends on the worst-case performance of $k = 3$. Recurrent structures, for which already a worst-case performance is determined, are indicated with a special symbol. The number within the symbol denotes the worst-case performance for that recurrent structure. Note that this example naturally extends to $k > 5$.

The above example gives the intuition behind the proof, the full proof shows why the worst-case performance is $4k - 5$, with k the number of vertices and $k \geq 3$. Before we use mathematical induction, we first need a small intermezzo to provide an expression for the worst-case performance. Let $P(n)$ be the worst-case number of LPs to be solved for a structure with n points. Here, n contains 2 'starting points' which can either be vertices or non-vertices in $\mathcal{Y}_N$ and $n - 2$ vertices. As we start with a given structure, we do not need to solve LPs for the 'starting points'. Then, $P(n)$ can be expressed as

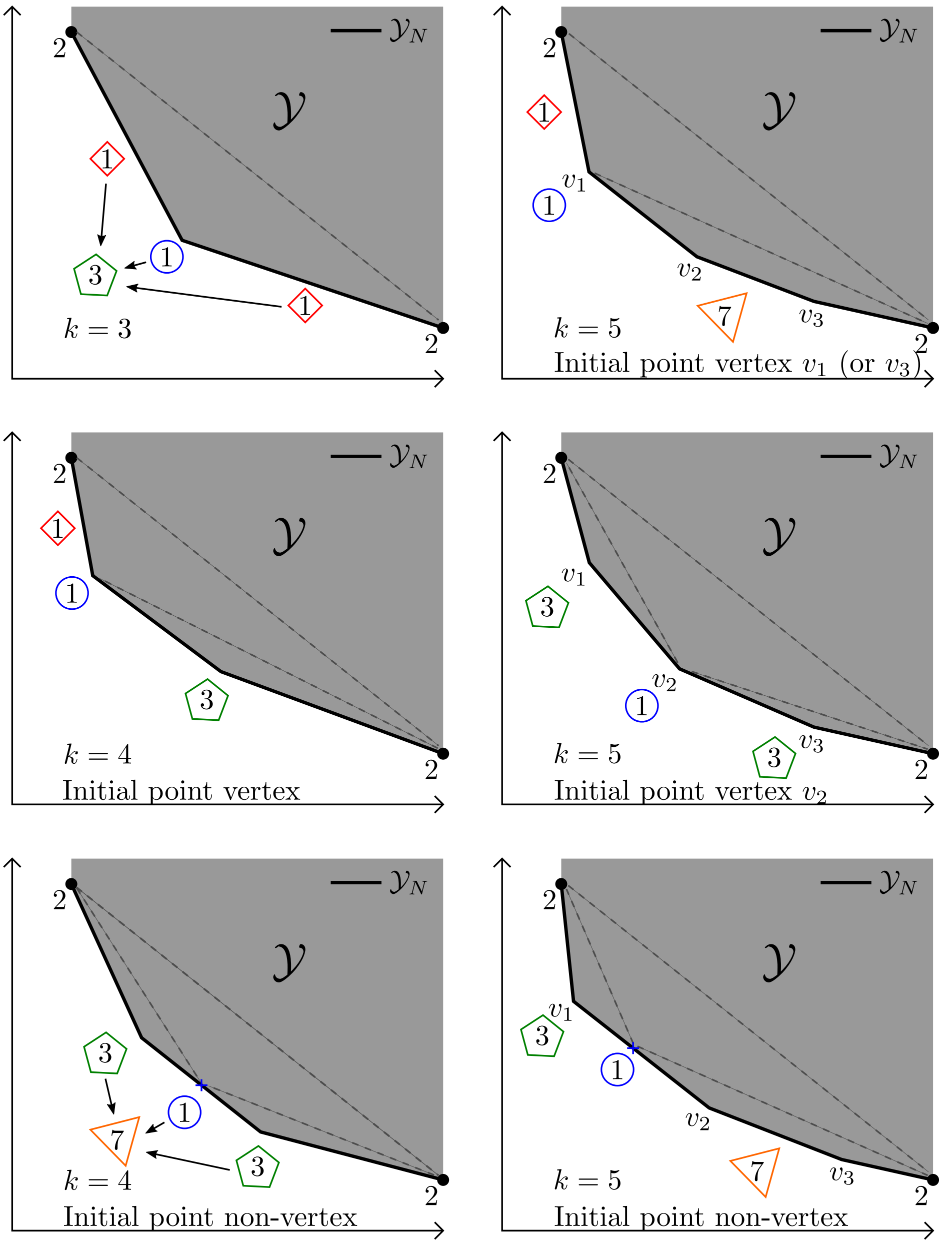

Figure S1. The worst-case performance in number of LPs solved for different numbers of vertices k. Recurrent structures, for which a worst-case performance is already known, are indicated with a pentagon and triangle where the number specifies the worst-case performance of that structure.

$$P(n) = \max_{\substack{i,j \text{ s.t.} \\ i,j \geq 3 \\ (i+j=n+1 \text{ or} \\ i+j=n+2)}} \{1 + P(i) + P(j)\} \tag{S1}$$

$$= 1 + \max_{\substack{i,j \text{ s.t.} \\ i,j \geq 3 \\ i+j=n+2}} \{P(i) + P(j)\} \tag{S2}$$

$$= 1 + \max_{\substack{i,j \text{ s.t.} \\ i=3,\ldots,n-1}} \{P(i) + P(n+2-i)\}\,. \tag{S3}$$

In Equation (S1) all possible splits of the substructures are considered, where the worst-case option is selected. In Equation (S2) the condition $i + j = n + 1$ can be removed as $P(n+1) \geq P(n)$. Equation (S3) combines $i, j \geq 3$ and $i + j = n + 2$ within the expression.

We now begin our mathematical induction proof, where we conjecture that

$$P(n) = 4n - 9 \quad , n \geq 3.$$

Let $n = 3$ be our base case, where on top of the starting points 3 additional LPs are solved. That is, 1 LP for finding the vertex on $\mathcal{Y}_N$ and 2 LPs to make sure that no vertex can be founds on both sides of this found vertex, which is visualized in Figure S1. As $1 + 1 + 1 = 3 = 4 \cdot 3 - 9$, our base case holds. For our induction hypothesis we assume that the above conjecture is correct for $P(i)$ with $i = 3, \ldots, n-1$, we now show it holds for $P(n)$ as well.

$$\begin{aligned} P(n) &= 1 + \max_{\substack{i,j \text{ s.t.} \\ i=3,\ldots,n-1}} \{P(i) + P(n+2-i)\} \\ &= 1 + \max_{\substack{i,j \text{ s.t.} \\ i=3,\ldots,n-1}} \{4i - 9 + 4(n+2-i) - 9\} \\ &= 1 + \max_{\substack{i,j \text{ s.t.} \\ i=3,\ldots,n-1}} \{4n - 10 + 4i - 4i)\} \\ &= 1 + \max_{\substack{i,j \text{ s.t.} \\ i=3,\ldots,n-1}} \{4n - 10\} \\ &= 4n - 9 \end{aligned}$$

As we initially have to solve 4 LPs to obtain efficient starting points, which are vertices, the worst-case performance is indeed $4 + P(k) = 4k - 5$ for $k \geq 3$ with k the number of vertices.

2 RESULTS FOR TRADE-OFF COSTS VERSUS NUTRITIONAL ADEQUACY WITH MINMAX ACHIEVEMENT FUNCTION

This is a supplement to Section 4.1. Here, we report the results for the adolescent female of 16-17 yrs when considering MinMax as the achievement function, where we look at the omnivore diet in particular. As the MinMax achievement function, which minimizes the deviation of the worst performing nutrient, still results in a linear model, the bi-objective approach as presented in Section 3.2 can be used to obtain all trade-offs between cost and nutritional adequacy.

Figure S2 shows the Pareto curves of all four diet types. The y-axis represents the daily cost of a diet and the x-axis describes the MinMax achievement function value. In this case the x-axis ranges from 0% to 100%, as no nutrient has a deviation larger than 100%. Each dot is a corner point found in the bi-objective algorithm, and each point on the line can be translated to a particular diet with corresponding cost and MinMax value. The leftmost and rightmost dots are the starting points of the algorithm. The leftmost dot of each diet type represents the diet with the least MinMax value possible, whereas the rightmost dot represents the cheapest diet adhering to all non-relaxed constraints, i.e. the exact energy intake and the food commodity constraints.

In line with our conclusions in Section 4.1, the figure shows that it is possible to create a diet without nutritional deviation for the omnivore, pescatarian and vegetarian diet type, as the MinMax value of their leftmost solution is 0%. This means that no nutrient has a deviation larger than 0%. Furthermore, the figure shows that for the vegan diet it is not possible to create a nutritionally adequate diet, as the leftmost dot has a 100% deviation. This indicates that at least one nutrient has a 100% deviation.

In Figure S3 a bar chart is presented which shows the diet composition of the omnivore diet given a certain cost. The x-axis represents the solution numbers, which are the vertices found from left to right in the Pareto curve of Figure S2, e.g. solutions 1 and 12 are the starting points. The left y-axis describes the cumulative number of gram included in a diet of a particular food commodity and the right y-axis shows the cost of a diet. The food commodities included in a solution are indicated with letters on the corresponding bar. Note that the right y-axis of Figure S3 matches with the y-axis of Figure S2, and thus for each solution number its MinMax value is known as well. Furthermore, note that the diet composition of solution 1 matches the diet composition of solution 1 in Figure 4B, as here the summed deviation and the MinMax achievement function both have the value 0, and that the diet composition of solution 12 matches the diet composition of solution 13 in Figure 4B, as the worst nutrient in Figure 4B in the rightmost solution has a 100% deviation as well.

In Figure S4 the nutritional content split by nutrient of each diet is shown given a particular cost. The x-axis represents the nutritional adequacy of a given nutrient relative to its lower limit, 100%, which is indicated with a red dotted line. In case applicable, the upper limit is indicated with a dark red dotted line. Thus, all values above the 100% and below the dark red dotted line indicate nutritional adequacy of that nutrient. The y-axis describes the daily cost and corresponds with the cost in Figure S2. The dots correspond to the corner points found on the Pareto curve.

As the achievement function focuses on the minimization of the worst performing nutrient, the MinMax achievement function does not give insights in how the other nutrients behave relative to the MinMax value. For example, a MinMax value of 50% could indicate that only one nutrient is deviating with 50%, but it could also mean that all nutrients deviate up to 50%. The summed deviation prevents this, as it takes the total deviation of all nutrients into account.

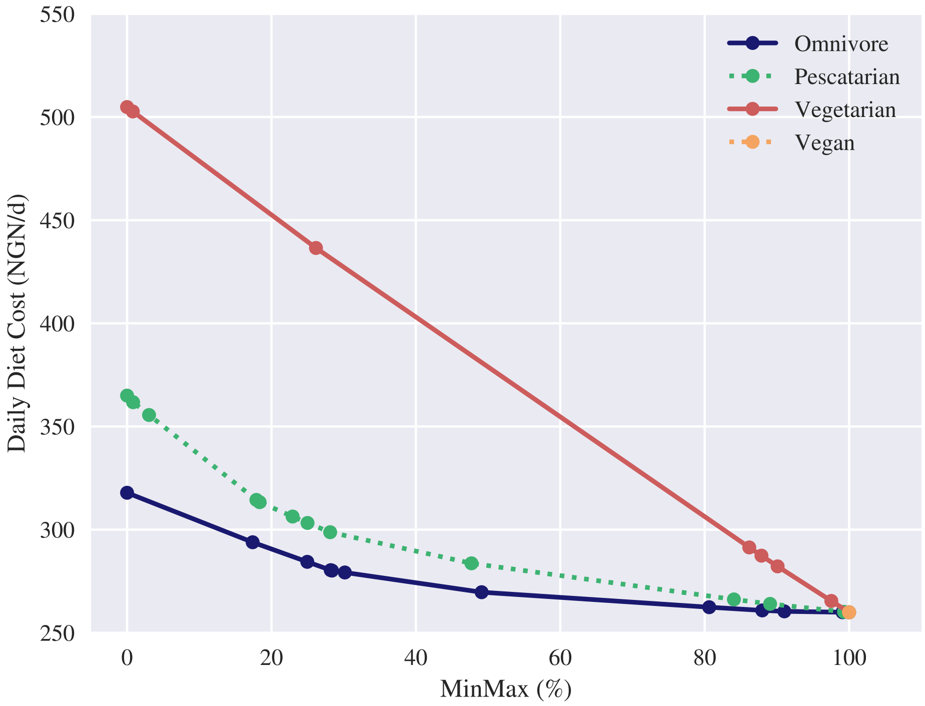

Figure S2. Pareto curve of the daily diet cost in Nigerian naira (NGN/d) versus the nutritional deviation measured with MinMax for an adolescent female of 16-17 yrs. in Ebonyi. Four diet types are considered: omnivore, pescatarian, vegetarian and vegan. Each dot is an efficient solution representing a trade-off between cost and nutritional adequacy.

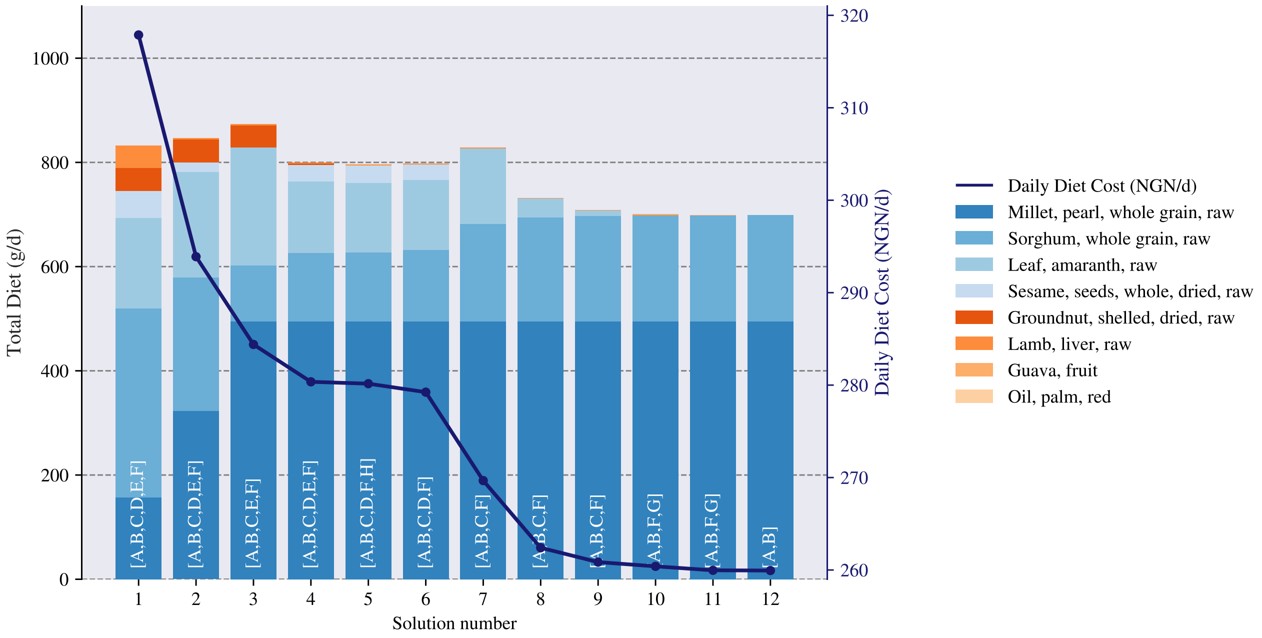

Figure S3. Daily diet composition corresponding to the trade-offs of the daily diet cost in Nigerian naira (NGN) versus nutritional deviation measured with MinMax for the omnivore diet for an adolescent female of 16-17 yrs. in Ebonyi. The left y-axis shows the total amount of a food commodity included in a particular diet in grams and the right y-axis shows the cost of the diet. The solution numbers on the x-axis correspond to the vertices/corner points found on the Pareto curve. The food commodities included in a particular diet are indicated with letters on the corresponding bar, where the length of the bar indicates the total daily grams of a food commodity included in the diet.

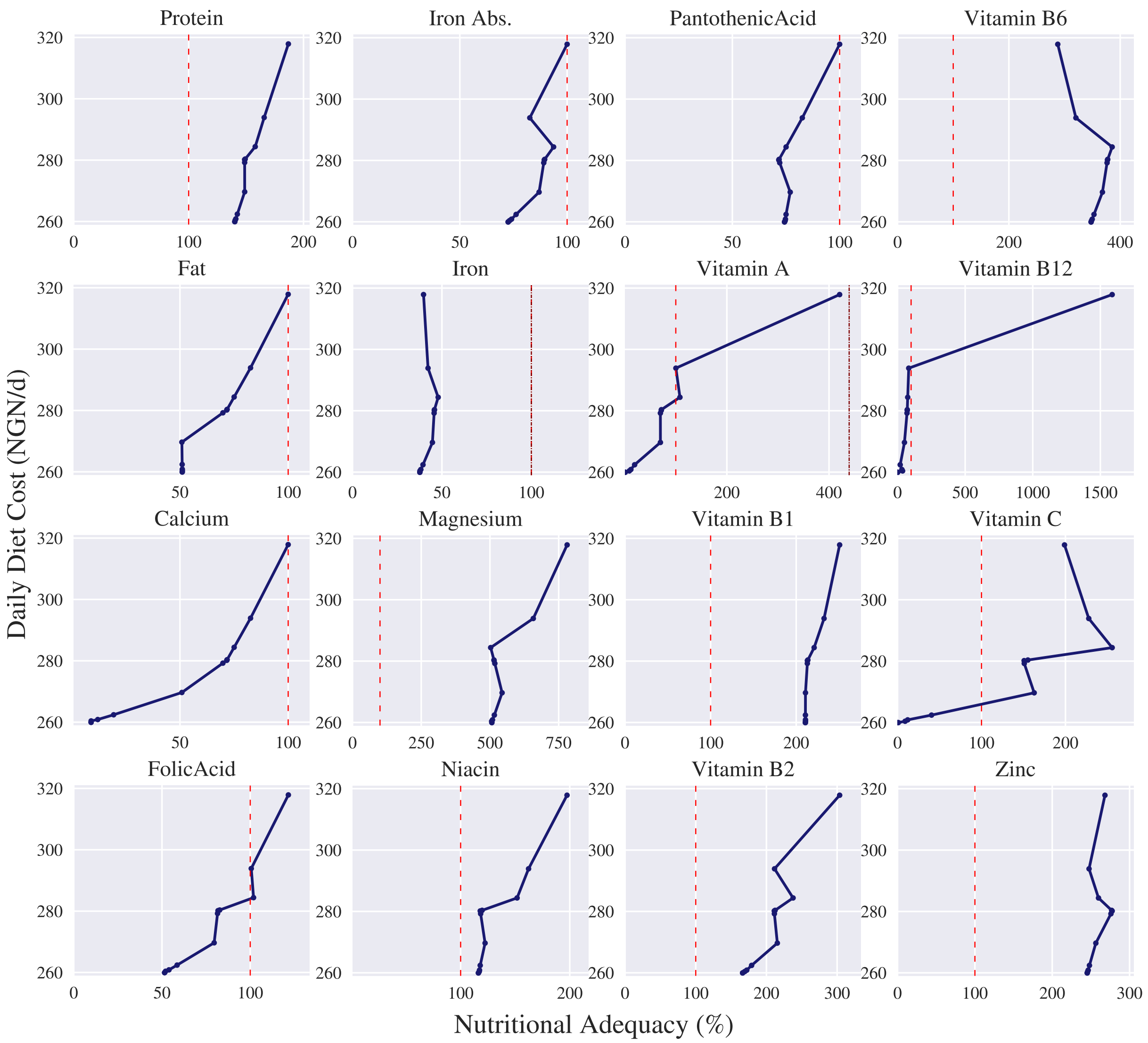

Figure S4. Nutritional adequacy per nutrient corresponding to the trade-offs of the daily diet cost in Nigerian naira (NGN/d) versus nutritional deviation measured with MinMax for the omnivore diet for an adolescent female of 16-17 yrs. in Ebonyi. For all nutrients, except iron, the adequacy is measured relative to their lower limit (100%). As iron only has an explicit upper limit, we measure its adequacy relative to its upper limit (100%). The daily cost of each dot corresponds to the daily cost on the Pareto curve. The lower limit of each nutrient is indicated with a red dotted line. If applicable, the upper limit is indicated with a dark red dotted line.

3 RESULTS FOR TRADE-OFF COSTS VERSUS NUTRITIONAL ADEQUACY WITH NUMBER OF UNMET CONSTRAINTS ACHIEVEMENT FUNCTION

This is a supplement to Section 4.1. Here, we report the results for the adolescent female of 16-17 yrs when considering the number of unmet constraints as the achievement function, where we look at the omnivore diet in particular. As using this achievement function results in a mixed-integer linear programming model, the bi-objective approach cannot be applied here. However, due to the structure of the problem it is possible to enumerate over all efficient points. To do so, we solve the DOP with the additional constraints imposed by (GP-Unmet), and with the constraint

$$\sum_{n \in \mathcal{N}} \underline{p}_n + \overline{p}_n \leq \beta,$$

where β is a parameter which states the maximum allowed number of unmet constraints. Allowing for a higher β results in more relaxation of the model and potentially lower cost. We can now solve the model for $\beta = 1, \ldots, |\mathcal{N}|$ and obtain the corresponding minimum cost.

Figure S5 shows all results when solving for different values of β for all four diet types. The y-axis represents the daily cost of a diet and the x-axis describes the maximum allowed number of unmet constraints β. In this case the x-axis ranges from 0 to 15, as up to 15 nutrients are allowed to deviate. Different from Figure 4A no line pieces are plotted, as a non-integer number of nutrients that does not meet the requirements is impossible. Note that the figure shows the maximum allowed number of unmet constraints, so in case β gives the same cost as $\beta - 1$ then β results in a so-called *weakly efficient point*. For example, using $\beta \geq 9$ results in the same cost. So, allowing for $\beta > 9$ results in weakly efficient points.

In Figure S6 a bar chart is presented which shows the diet composition of the omnivore diet given a certain cost. The x-axis corresponds with the values for $\beta = 1, \ldots, 15$. The left y-axis describes the cumulative number of gram added to a diet of a particular food commodity and the right y-axis shows the cost of a diet. The food commodities included in a solution are indicated with letters on the corresponding bar. Note that solution 1 corresponds with solution 1 in Figure 4B, as in both solutions no nutrients are deviating.

In Figure S7 the nutritional content split by nutrient of a diet is shown given a particular cost. The x-axis represents the nutritional adequacy of a given nutrient relative to its lower limit, 100%, which is indicated with a red dotted line. In case applicable, the upper limit is indicated with a dark red dotted line. Thus, all values above the 100% and below the dark red dotted line indicate nutritional adequacy of that nutrient. The y-axis describes the daily cost and corresponds with the cost in Figure S5. The dots correspond to the solutions found in Figure S5. Note that dots of different solutions may overlap, which is especially the case for $\beta \geq 8$. Furthermore, note that solution 9 of Figure S6 corresponds with solution 13 in Figure 4B, as the same nutrients are deviating and the diet composition is the same as well.

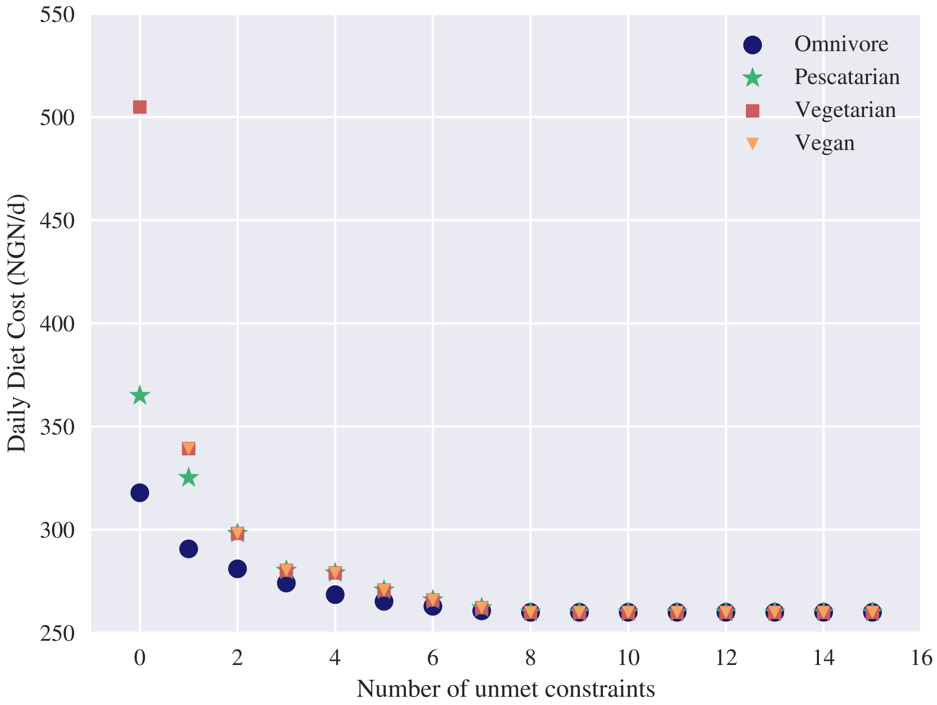

Figure S5. All combinations of the daily diet cost in Nigerian naira (NGN/d) versus the maximum allowed number of unmet nutritional constraints for an adolescent female of 16-17 yrs. in Ebonyi. Four diet types are considered: omnivore, pescatarian, vegetarian and vegan. A solution is efficient if no other solution to its left of the same diet type has the same cost.

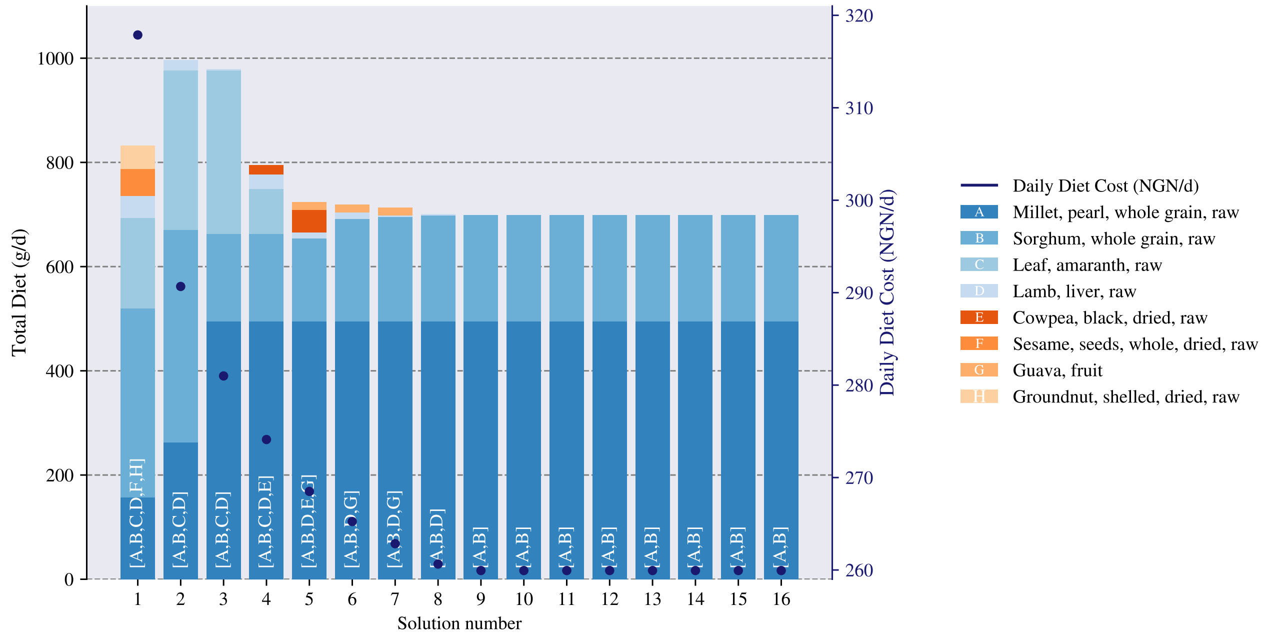

Figure S6. Diet composition corresponding to the trade-offs of the daily diet cost in Nigerian naira (NGN) versus nutritional deviation with the maximum allowed number of unmet constraints for the omnivore diet for an adolescent female of 16-17 yrs. in Ebonyi. The left y-axis shows the total amount of a food commodity included in a particular diet in grams and the right y-axis shows the cost of the diet. The solution numbers on the x-axis correspond to the maximum number of nutrients allowed to deviate plus one. The food commodities included in a particular diet are indicated with letters on the corresponding bar, where the length of the bar indicates the total daily grams of a food commodity included in the diet.

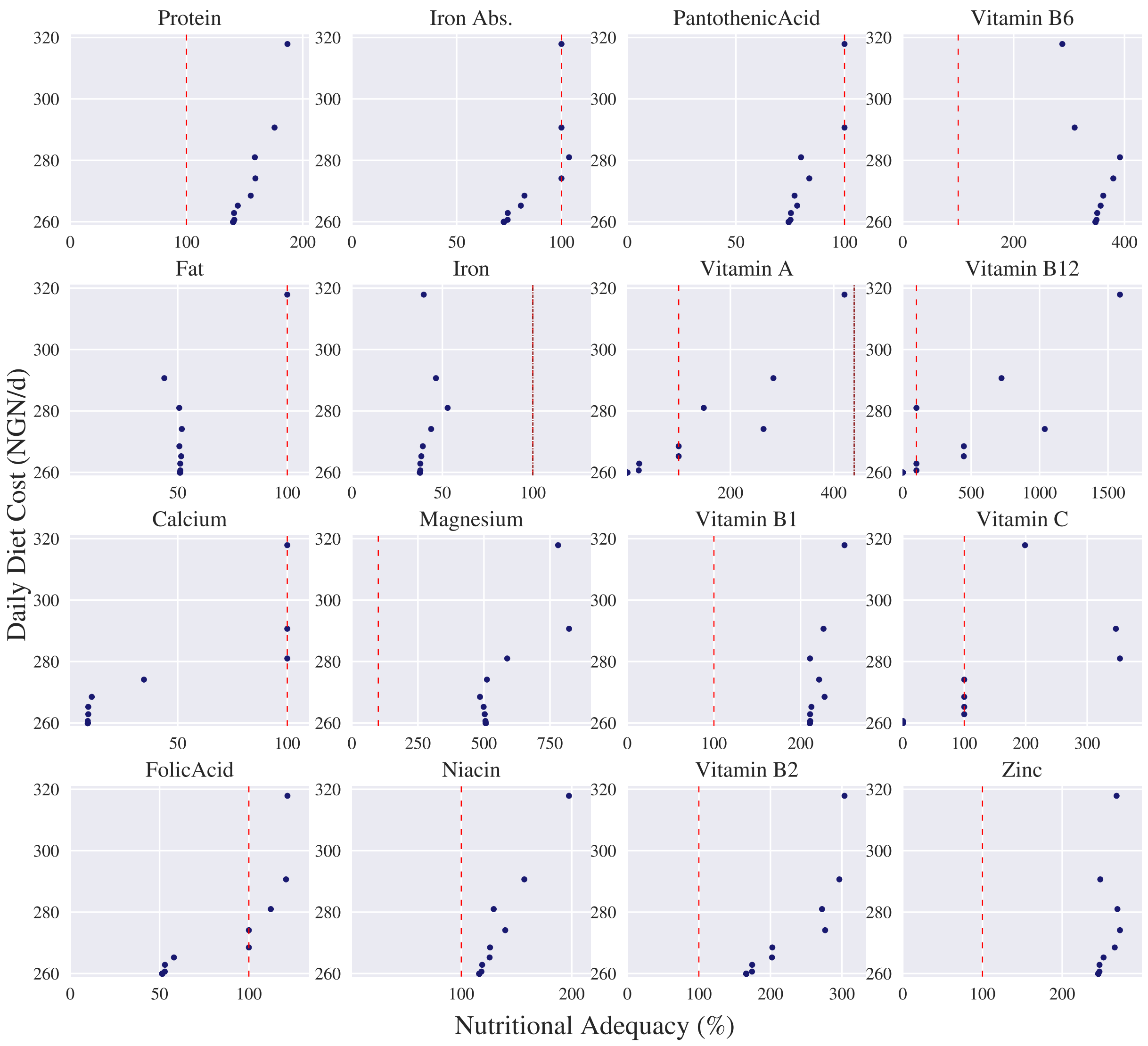

Figure S7. Nutritional adequacy per nutrient corresponding to the trade-offs of the daily diet cost in Nigerian naira (NGN) versus nutritional deviation measured with the number of unmet constraints for the omnivore diet for an adolescent female of 16-17 yrs. in Ebonyi. For all nutrients, except iron, the adequacy is measured relative to their lower limit (100%). As iron only has an explicit upper limit, we measure its adequacy relative to its upper limit (100%). The daily cost of each dot corresponds to the daily cost on the Pareto curve. The lower limit of each nutrient is indicated with a red dotted line. If applicable, the upper limit is indicated with a dark red dotted line.

4 EXPLANATION MORE-FOR-LESS PARADOX

In general, it is possible to check some conditions to understand whether the more-for-less paradox has taken place. To define these conditions (53), the LP is structured in the following way:

$$\begin{aligned} \min c^\top x &= \sum_{i=1}^{n} c_i x_i \\ \text{s.t. } Ax &= b \\ x &\geq 0, \end{aligned}$$

where x is an $n \times 1$ vector consisting of decision variables, c an $n \times 1$ cost vector, b an $m \times 1$ coefficient vector and A an $m \times n$ coefficient matrix. Note that it is always possible to rewrite a linear program to this standard format by introducing slack variables. In case matrix A has full rank and the dual of constraint $j \in \{1, \ldots, m\}$ is negative, then the objective can be improved by increasing b_j without altering the other coefficients.

This phenomenon has a straightforward cause which can be understood by looking at the conditions more closely (53). First, note that the dual variable of constraint $j \in \{1, \ldots, m\}$ is unbounded due to the equality constraints. So, negative dual variables are allowed in the dual formulation. Secondly, note that the found optimal solution is unique because of the full rank of A and thus no other optimal solution exists. Assume we have now found the (unique) optimal solution for the above problem in which at least one negative dual variable is found. This optimal solution is not optimal for the problem where the equality constraint is adjusted to a $\geq$ constraint: in which case all the corresponding dual variables should be non-negative. Thus, the found solution is not dual feasible for the problem with inequality constraints, whereas it is still primal feasible. This indicates that the found solution that is optimal for the model with equality constraints is nonoptimal for the model with inequality constraints. Returning to our example in Section 4.2.1, we find that the dual of Equation (4.2) is indeed negative. So, we can increase the energy requirement within an acceptable range while improving on the cost of the diet. In our example, it is even possible to still lower the cost by both increasing the energy requirement — which thus lowers the cost — and the folic acid intake, which normally increases the cost, as it is a $\geq$ constraint.
